# Prioritizing Metabolic Gene Regulators through Multi-Omic Network Integration in Maize

**DOI:** 10.1101/2024.02.26.582075

**Published:** 2024-02-27

**Authors:** Fabio Gomez-Cano, Jonas Rodriguez, Peng Zhou, Yi-Hsuan Chu, Erika Magnusson, Lina Gomez-Cano, Arjun Krishnan, Nathan M Springer, Natalia de Leon, Erich Grotewold

**Affiliations:** 1 Department of Biochemistry and Molecular Biology, Michigan State University, East Lansing, MI 48824-6473, USA.; 2 Department of Plant and Agroecosystem Sciences, University of Wisconsin Madison, Madison, WI 53706, USA; 3 Department of Plant and Microbial Biology, University of Minnesota, Saint Paul, MN 55108; 4 Department of Biomedical Informatics, University of Colorado Anschutz Medical Campus, Aurora, CO 80045, USA; 5 Current address: Global Breeding, Bayer Crop Sciences, Chesterfield MO 63017, USA; 6 Current address: Department of Molecular, Cellular, and Development Biology, University of Michigan, Ann Arbor, MI, 48109, USA.

**Keywords:** Transcription factor, PDI, eQTL, Co-expression, Gene regulation

## Abstract

Elucidating gene regulatory networks (GRNs) is a major area of study within plant systems biology. Phenotypic traits are intricately linked to specific gene expression profiles. These expression patterns arise primarily from regulatory connections between sets of transcription factors (TFs) and their target genes. In this study, we integrated publicly available co-expression networks derived from more than 6,000 RNA-seq samples, 283 protein-DNA interaction assays, and 16 million of SNPs used to identify expression quantitative loci (eQTL), to construct TF-target networks. In total, we analyzed ~4.6M interactions to generate four distinct types of TF-target networks: co-expression, protein-DNA interaction (PDI), *trans-*expression quantitative loci (*trans*-eQTL), and *cis*-eQTL combined with PDIs. To improve the functional annotation of TFs based on its target genes, we implemented three different strategies to integrate these four types of networks. We subsequently evaluated the effectiveness of our method through loss-of function mutant and random networks. The multi-network integration allowed us to identify transcriptional regulators of hormone-, metabolic- and development-related processes. Finally, using the topological properties of the fully integrated network, we identified potentially functional redundant TF paralogs. Our findings retrieved functions previously documented for numerous TFs and revealed novel functions that are crucial for informing the design of future experiments. The approach here-described lays the foundation for the integration of multi-omic datasets in maize and other plant systems.

## Introduction

Like other organisms, plant cells use interconnected molecular networks that collaboratively coordinate every cellular process, from cell division to metabolite synthesis and adaptation. Among these, the regulatory network plays a unique role by controlling the expression of every gene in the cell. Function that is carried out mainly by transcription factor (TF) proteins (Lambert et al., 2018). The transcriptional regulation of genes requires direct protein-DNA interactions (PDI) between TFs and specific *cis*-regulatory elements (CREs) located in proximal (promoters) or distal (enhancers/silencers) regulatory regions of the corresponding target gene (Schmitz et al., 2022). Furthermore, TFs can also regulate gene expression indirectly by engaging in protein-protein interactions (PPI) and being tethered to DNA through other PDIs. Together, collections of PDIs form highly interconnected gene regulatory networks (GRNs). Overall, the architecture of GRNs is elucidated using combinations of gene- and TF-centered approaches (Arda and Walhout, 2010; Mejia-Guerra et al., 2012; Yang et al., 2016). Common gene-centered methods include yeast one-hybrid (Y1H) assays (Arda and Walhout, 2010; Yang et al., 2016). TF-centered strategies involve techniques like chromatin immunoprecipitation followed by sequencing (ChIP-seq) for *in vivo* PDI discovery and DNA affinity purification sequencing (DAP-Seq) for *in vitro* analyses (Yang et al., 2016; O’Malley et al., 2016). However, ChIP-seq has limitations in scalability to multiple TFs, while DAP-seq captures interactions that lack of native chromatin environment (Yang et al., 2016). The organization of GRNs has implications for phenotypic variation (Deplancke et al., 2006), plant responses to abiotic and biotic stress (Nakashima et al., 2014; Birkenbihl et al., 2017; Sun et al., 2022), development (Marand et al., 2023), speciation (Mack and Nachman, 2017), as well as adaptation and diversification (Mack and Nachman, 2017; Bowles et al., 2020; Marand et al., 2023), among others. Hence, comprehending the structure and dynamics of GRNs offers a valuable avenue and strategy for manipulating desired phenotypes.

Maize holds great agricultural significance due to its broad adaptation and wide range of applications (Erenstein et al., 2022). Part of what provides maize with its versatility is its extraordinary metabolic diversity (Riedelsheimer et al., 2012; Wen et al., 2014, 2016; Zhou et al., 2019), which is underpinned by its genetic diversity (Schnable et al., 2009; Hufford et al., 2021; McMullen et al., 2009), and varies depending on endogenous variables (e.g., organs or development stages) (Zhou et al., 2019) and environmental factors (Wen et al., 2014; Kusmec et al., 2017). Similar to other plant and animal systems, quantitative traits in maize are genetically complex, illustrated by the large number of loci associated with a single trait and the minor contribution of any given association to the trait’s variance (Riedelsheimer et al., 2012; Wen et al., 2014, 2016; Xiao et al., 2017; Mazaheri et al., 2019; Zhou et al., 2019). Hence, elucidating the molecular mechanisms underlying quantitative traits faces two primary obstacles when only simple trait-loci associations are considered. These obstacles include the involvement of multiple genes governing a single phenotypic outcome and the impact of additional genetic elements that determine and modulate the genetic contribution to phenotypic variations.

A distinctive genomic characteristic of maize is a recent whole genome duplication (WGD) event, which occured~5–12 Mya, making maize an ancient allotetraploid (Wei et al., 2007). Maize has an abundance of tandem duplicated genes (Kono et al., 2018), and is highly-enriched in transposable elements (~85% of genome sequence) (Schnable et al., 2009). Furthermore, the WGD event resulted in the formation of two subgenomes (*maize1* and *maize2*), exhibiting unequal gene loss and expression patterns, primarily driven by the subgenome with a lower fractionation rate (Schnable et al., 2011). The dominant subgenome *maize1* was shown to have a larger contribution to phenotypic variations (Renny-Byfield et al., 2017). Nevertheless, the precise molecular mechanisms underlying the asymmetric contributions of each subgenome remain largely unknown and are likely orchestrated at multiple molecular levels, including regulation, signaling, and interactome level, as evidenced by examining co-expression and multi-network comparisons of homeologs (Li et al., 2016; Han et al., 2023). Understanding these mechanisms holds significant implications for modeling, prioritizing, and unraveling the principal factors behind agriculturally relevant traits, while also advancing our fundamental comprehension of maize evolution.

Recently, generation of multi-omic datasets have gained traction as a means to understand the complexity of genetic and phenotypic variation observed in biological systems, offering insights at various levels of biological organization (Tolani et al., 2021). Integrating these diverse datasets is an evolving field, with conceptual integration (overlapping observations), statistical integration, network-based integration, and machine learning-based integration being the four main approaches used (Depuydt et al., 2023). In maize, like other model organisms, technological advancements have also favored the rapid generation of multi-omic data. These data encompass various molecular layers at different scales including TF binding profiles (Galli et al., 2018; Ricci et al., 2019; Tu et al., 2020), accessible chromatin regions (ACRs) (Rodgers-Melnick et al., 2016; Ricci et al., 2019; Marand et al., 2021), expression and co-expression atlases (Sekhon et al., 2011; Stelpflug et al., 2016; Hoopes et al., 2019; Zhou et al., 2020), and population-level transcriptomic, proteomic, and metabolic information (Li et al., 2013; Wen et al., 2014, 2016; Kremling et al., 2018; Zhou et al., 2019; Mazaheri et al., 2019; Shrestha et al., 2022). Consequently, the growth generation of multi-omic datasets has been a growing focus on the establishment of strategies for their integrating (Liu et al., 2016; Walley et al., 2016; Wen et al., 2016; Jin et al., 2017; de Abreu E Lima et al., 2018; Lee et al., 2019; Schaefer et al., 2018; Wen et al., 2018). However, most integration efforts primarily involve the verification of each layer with one another (i.e., conceptual integration) (Depuydt et al., 2023). There are a few exceptions where the layers are leveraged to enhance the integration (Schaefer et al., 2018; Yang et al., 2022) or to learn from their combined information (Han et al., 2023). Therefore, emphasizing the need for a comprehensive assessment of integration strategies and its effectiveness to prioritize gene-specific processes.

In this study, we analyzed genetic and gene expression variation across 304 maize inbred lines. We utilized data from over 300 publicly available ChIP- and DAP-seq experiments, along with 45 previously analyzed co-expression networks (Zhou et al., 2020). Thus, we aim to define a robust strategy to integrate the corresponding datasets and then use the corresponding strategy to identify maize metabolic gene regulators. Combining these datasets, we built four molecular networks and employed three integration methods with the goal of annotating TFs based on their predicted target genes. We combined previously published expression datasets from loss-of-functions (aka., knockouts) and randomly generated interactions as strategies to evaluate the different functional annotation strategies. This enabled us to pinpoint the integration strategy that yielded functional predictions closely resembling those observed in knockout assays, while simultaneously reducing the likelihood of random predictions. We provide evidence that these predictions recovered TFs and previously associated biological processes. The compiled predictions enabled the creation of a TF-process association list, which, when combined with TF-target networks, facilitated the identification of regulators for different metabolism pathways, including abscisic acid (ABA), lipid, phenylpropanoid, as well as leaf-development related processes. Finally, we demonstrated that integrating all networks into a unified, combined network facilitated the generation of embedding which recovers patterns of connectivity within the comprehensive network. This approach enabled us to identify potentially redundant genes. Collectively, these findings offer a rich integration of TF-process associations, laying the foundation for future network-based functional prediction work in maize, and facilitating the linkage of previously identified genetic markers with clusters of functionally associated genes, utilizing connectivity patterns within the networks.

## Results

### Construction of a maize regulatory network based on multiple layers of information.

**Figure 1.**
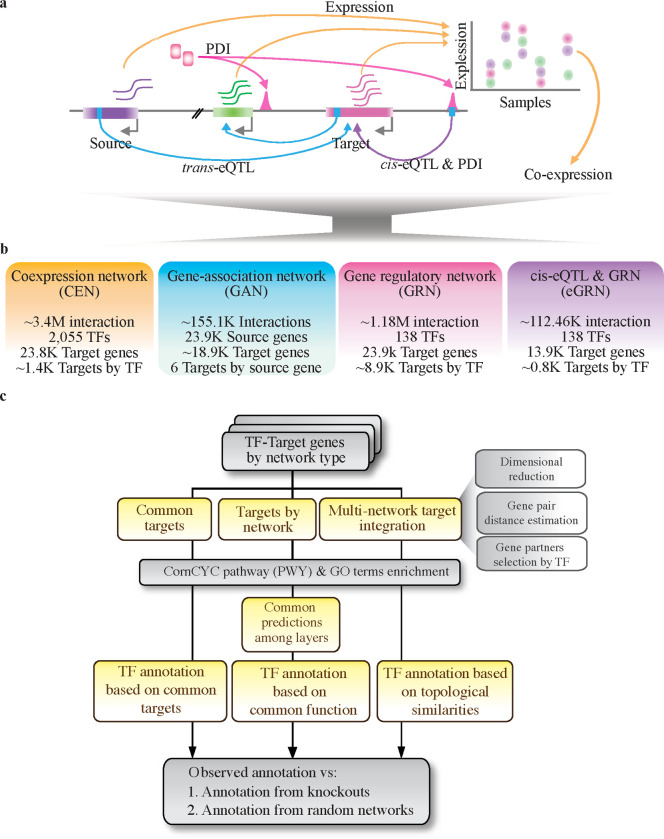
Construction of maize gene regulatory network based on multiple data types. **a**. Model indicating the different types of TF-gene associations used to define the network types analyzed in this work. **b**. Summary of the metrics of the four types of network layers. **c**. Schematic representation of the pipeline implemented to annotate and evaluate the corresponding functional predictions.

To build a multi-layer TF-function association network, we collected previously published co-expression networks, single-nucleotide polymorphisms (SNPs), and reanalyzed publicly available expression, DAP-seq, and ChIP-seq datasets from maize. We included several co-expression networks, genetic variation data for 304 maize inbred lines (Table S1), and 283 DNA-binding assays (DAP-seq and ChIP-seq) associated with 142 TFs (Table S2) (Figure 1a). In total, we identified ~3.4M TF-gene associations derived from the co-expression networks (CENs), ~155.1K from the gene association network based on *trans*-eQTL (GAN), ~1.18M in the GRN, and another 112.46K from the *cis*-eQTLs overlapped with GRN interactions (eGRN) (Figure 1b). Construction details for the corresponding networks are described below.

#### Co-expression network (CEN).

To build the CEN layer, we started by collecting previously published 45 CENs (Zhou et al., 2020), and added an additional network constructed with a subset of expression datasets associated with 304 inbred lines from the Wisconsin Diversity (WiDiv) panel (Mazaheri et al., 2019). The 304 lines were selected based on availability of high-density whole genome sequencing derived SNPs (Bukowski et al., 2018), following consistent methods with previously reported CENs (Zhou et al., 2020) (see Methods). Thus, in total, we utilized 46 different co-expression networks to define the TF-target CEN layer (Table S3). Each network was reduced to only maize genes in synteny with *Sorghum bicolor* (Schnable, 2019) to avoid potential bias towards non-functional genes when conducting gene enrichment analyses. The syntenic gene filter was also applied to all other network types (*i.e*., GRN, eGRN, and GAN). On average, we found 1,055 TFs per CEN (Figure S1a) which had - on average - 74 predicted target genes each (Figure S1b). Combining all 46 CENs, we identified ~3.4M TF-target associations involving 1,852 TFs and 23,788 targets (on average, ~1,350 targets per TF; targets can include other TF genes). Some TFs had orders of magnitude more targets than the average TF (Figure S1b). For example, ABI3VP1–7 (ABI7) and C2C2-CO-like-8 (COL8) showed >400 targets in five and four CENs, respectively (Figure S1b); or COL13 and bHLH127, which in total showed > 6,000 targets each (Figure S1c).

#### *Gene association network* based on *trans*-*e*QTLs (*GAN)*.

This layer was built based on *trans*-eQTLs identified in eight distinct tissues encompassing several developmental stages. Overall, after quality control and data preprocessing (see Methods), we tested between 15.5M and 16.7M SNPs against the expression of 15.3K to 26.4K genes across the eight tissue types (Table S4). Thus, after discarding non-significant eQTLs (See Methods) and non-syntenic genes (Schnable, 2019), we obtained a total of ~22.9M eQTL-gene associations including ~10M different SNPs and ~26.4k target genes. These associations were classified as *trans/cis*-eQTL, *cis*-eQTL, *trans*-eQTL, unassigned eQTL depending on the distance between each eQTL and its corresponding target gene (Figure S2a). Finally, we also include another category, *cis*-eQTLt, which describes eQTL co-localized with its target genes (Figure S2a, yellow collection). Under this classification schema, we identified 10.2M unassigned eQTL, 6.7M *trans*-eQTL, 1.20M *trans/cis*-eQTL, 1.18M *cis*-eQTLt, and 3.5M *cis*-eQTL (Figure S2a). Within them, *trans*-eQTLs (eQTLs overlapped with annotated genes and located >50 kbs far away from their corresponding target genes) were used to define the GAN. Specifically, the “source gene” was described as a gene that is co-localized with an eQTL, while the “target gene” is the gene whose expression was explained by the corresponding eQTL (Figure S2a, gene blue and gene yellow, respectively).

After removing redundant associations (*i.e.,* multiple eQTL supporting the same gene-to-gene connection), the resulting GAN harbored ~155k associations, including 23.9K source and 18.9K target genes. To further interrogate the genes captured on the predicted GAN, we classified source and target genes into five functional categories including transcription factors (TFs), co-regulatory factors (CoReg), transcriptional mediator complex proteins (mediator), kinases, enzymes, and others (Table S5) (Yilmaz et al., 2009; Zheng et al., 2016; Mathur et al., 2011). The “Kinase” and “Enzyme” classes were the top two with the highest number of target genes, surpassing the “TF” class (Figure S2b). Similarly, the “Enzyme” class was the most frequent target gene class followed by “Mediator” and co-regulators (“CoReg”) (Figure S2c). We counted the interaction frequency between the corresponding classes, and after “Other”, “Enzyme” was the functional class with more interactions (13.7K) (Figure S2d), highlighting “Enzymes” as one of the functional classes most interconnected within the predicted GAN. Finally, we also noted that the GAN recovered gene-gene interactions that captured both typical TF-target interactions, but also PPIs. An example is provided by TF HSF20 which showed 354 targets, including 27 genes previously reported as heat-response related genes (Zhou et al., 2021), as well as five known physical interactors of HSF20 (Table S6) (Zhu et al., 2016). Altogether, highlights an unexplored set of regulatory connections among genes at several hierarchical levels.

#### *Gene-regulatory network (GRN),* and *cis-eQTLs* overlapped with *GRN interactions (eGRN)*.

To construct the GRN, we collected and reanalyzed 283 PDI experiments associated with 142 different TFs. All of the reanalyzed datasets corresponded to TF-centered approaches, including 245 ChIP-seq experiments in maize and 38 DAP-seq. We used a single data analysis pipeline to process all PDI assays to control for technical variations in the analysis (See Methods). On average, we obtained ~52k TF binding peaks per TF which, in total, represented ~7.6M PDIs (Table S7). The large majority of the predicted peaks were contributed by 215 protoplast ChIP-seq experiments (Tu et al., 2020), which represented 75% of the data analyzed (on average, ~55k peaks per TF) (Figure S3a). To identify high-confidence peaks, we applied two filtering criteria. First, we gathered accessible chromatin regions (ACRs) from the recently published single-nuclei ATAC-seq (snATAC-seq) atlas (Marand et al., 2021), retaining only TF peaks that overlapped with ACRs. Therefore, we compared all the DAP-seq and ChIP-seq datasets to a shared open chromatin regulatory maize space. Second, we removed peaks with low counts per million (CPM) (as defined by a Z-score ≤ −0.5) for each PDI assay. Overall, we eliminated ~3.8M peaks using the ACR criteria and additional ~1.1M peaks with the CPM cut-off (Figure S3b, c). As expected, most of the eliminated peaks were derived from DAP-seq assays (Figure S3b, c). Comparing low-coverage and ACR co-location peaks and their distance to the closest annotated transcription start site (TSS), we found that peaks with the highest Z-values mapped largely to ACRs near TSSs (~10 kbs to each side of the TSS) (Figure S3d). These last patterns were observed in all data types (DAP-, ChIP-, and pChIP-seq), supporting the biological relevance of the high-confidence peaks retained. After filtering, we ended with a set of ~3.4M peaks that were used for downstream analyses.

To define target genes, we integrated the peak-TSS distance and their overlap with *cis*-eQTLs (declared when a peak summit and a *cis*-eQTL were ≤ 20 bp away). Integrating these metrics, we categorized the peaks into three types when they were in close proximity to transcription start sites (TSSs), defined as ≤ 3 kb, and two types when they were further away (> 3 kb and ≤ 50 kb). Specifically, peaks within proximity (≤ 3 kb) of the TSS were classified as follows: peaks lacking cis-eQTL support (light purple peaks, see Figure S3e), peaks with cis-eQTL support and consistent target prediction (light green peaks, see Figure S3e), and peaks with cis-eQTL support but discordant target prediction (yellow peaks, see Figure S3e). These categories represented the 54.9%, 1.9%, and 0.1% of the total analyzed peaks, respectively. Similarly, peaks located far away were classified as peaks with (3.3%) and without (39.5%) *cis*-eQTL support (Figure S3e; light blue and gray peaks, respectively). Overall, we did not observe differences in peak categories among PDI data types (Figure S3e, bottom panel). Thus, after discarding peaks located far away and without *cis*-eQTL support, we built a GRN and *cis*-eQTL supporting GRN (eGRN) combining all peaks by TF irrespectively of the PDI source. In total, we captured ~1.12M (GRN) and ~1123.46K (eGRN) TF-target interactions, including 138 TFs and ~23.9K and 13.9K target genes, respectively (Figure 1a, b).

### TF functional annotation

One significant contrast among the constructed networks lies in the varying count of TFs and their corresponding target or associated genes (herein interchangeably referred to as target genes), posing a challenge for inter-layer comparisons. For instance, while all four networks feature 111 TFs with at least one associated target gene (see Figure S4a, b), this count dwindles drastically to just 17 TFs when considering TFs with at least ten distinct target genes per network (see Figure S4a, c). This reduction is largely caused by the low number of predicted targets on the GAN layer (on average, ~6.5 targets by gene). Therefore, we implemented three different strategies (*common interactions, common integrations,* and *network-based*) to functionally annotate the TFs present in the corresponding networks. In all three approaches, the annotation was performed based on enrichment of metabolic pathways (PWYs) (Andorf et al., 2016) and GO terms (Wimalanathan et al., 2018) (Figure 1c) (See Methods). Briefly, the most conservative *common interactions* approach assumes that only common TF-target interactions between layers (i.e., GAN, GRN, eGRN, and CEN networks) capture true targets of the corresponding TF, and by extension its function. *Common function* assumes that a TF function is most accurately captured by those functions commonly enriched across different network types. Thus, it prioritizes functions commonly enriched for the corresponding TF across layers. Finally, *network-based* combines all layers to then extract topological properties for each gene. It assumes that each interaction type bore equally valid information about the function of the corresponding TFs. Specifically, it combines all four layers (GAN, CEN, eGRN, and GRN) creating a dense combined network from which to extract physical parameters - embeddings - from each gene in the combined network (See Methods). The transformation of the networks into a matrix of genes and embeddings allows the grouping of genes based on the similarity of their embeddings. Here, we used the mutual rank of the mutual information as the metric to identify highly similar genes in the embedding matrix, to subsequently test for enrichment with PWYs and GO terms between the corresponding genes. The topologically informed strategy allowed us to functionally annotate TFs independently of the number of target genes predicted at the individual network layers.

**Figure 2.**
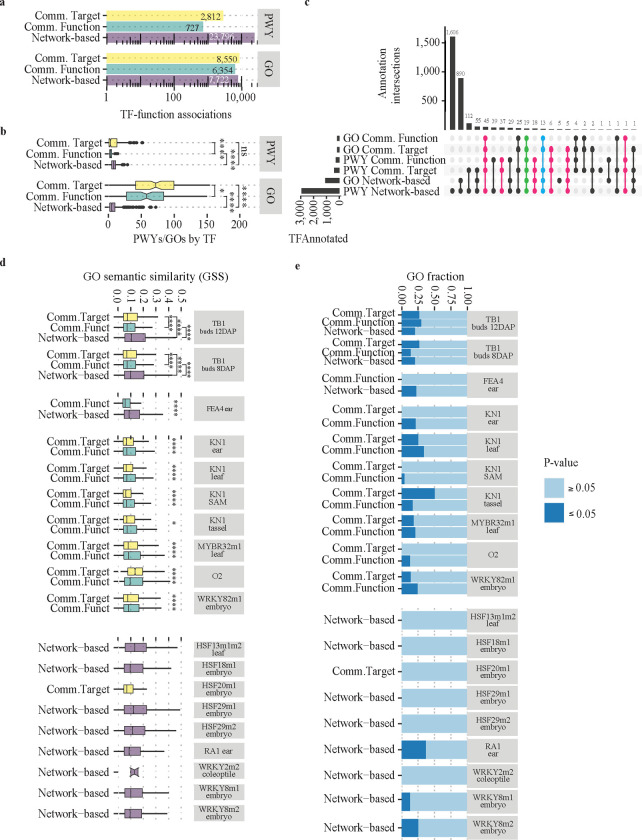
Annotation and evaluation of TF functional annotation by contrasting predictions with knockout assays and random networks. Total PWYs and GO terms predicted per integration method after combining all TFs predictions (**a**) and per TF (**b**). **c**. Upset plot comparing total TFs annotated by each method and annotation system. Colors indicate the groups of TFs functionally annotated by all three methods by enrichment with PWYs (fuchsia), GO terms (blue), and both PWYs and GO terms (green). Black groups indicate TFs annotated by at least one of the methods and annotation systems. **d**. Boxplot of the GO semantic similarity for the top 10 most similar GO terms observed in knockout assays for each of the predicted GO terms per TF and methods. **e**. Stacked barplot indicating the fraction of the GO terms predicted and significantly enriched - by GSEA analysis - in the knockouts. **f**. Violin plot showing the fraction of random networks with at least one significant (FDR ≤ 0.1, Fisher exact test) GO term by TF. Asterisks indicate P-value significance (*: p ≤ 0.05, **: 633 p ≤ 0.01, ***: p ≤ 0.001, ****: p ≤ 0.0001, two-sided t-test). “TFm” denotes multiple mutant lines for the same TF.

Extended descriptions of the functional annotation *Common interactions* and *Common function* are presented in supplemental material (Supplemental data 1). Briefly, using *Common interactions*, we found 11,362 significant associations between TF and biological processes (TF-process), including 2,812 associations between TFs and PWY (TF-PWY) and 8,550 between TFs and GOs (TF-GO) [False Discovery Rate (FDR) ≤ 0.1, Fisher’s Exact Test] (Figure 2a). On average, we identified ~8 PWYs and ~80 GO terms by TF (Figure 2b, boxplot yellow). Combining the PWY and GO term results, we annotated 347 TFs, out of which 235 TFs showed enrichment only in the PWYs analysis (Table S8). The remaining 112 TFs showed enrichment with both PWYs and GO terms (Figure 2c). Continuation with *Common function,* in total, we find 7,081 TF-process annotations (727 TF-PWY and 6,354 TF-GO) (Table S8) (Figure 2a). On average, this corresponds to 3.5 different PWYs and 57.7 different GO term associations per TF (Figure 2b). In terms of TFs, these associations encompass annotations for 204 TFs through PWY enrichment and 110 TFs through GO term enrichment (Figure 2c).

#### Network-based.

We combined all four layers and scaled the interaction frequencies. Using the scaled interaction frequencies, we identified low-dimensional representations (embeddings) for each gene/node in the combined network that works as descriptors of the corresponding gene (Figure S6a) (See Methods). The combined network included 4.6M interactions associated with 36.4K genes. Unlike the previous two strategies, this method generated an equal number of embeddings for each gene in the network, thus uncovering genes with similar properties, including TFs present in the CEN and/or GAN layers without data on the GRN/eGRN layers. The similarity of the embeddings between genes was used as a metric to assess the wiring similarities between genes within the combined network (See Methods) (Figure S6a). On average, we found 235 highly similar genes per TF [Distance (D), ≤ 0.05, See Methods] (Figure S6b). As in previous approaches, we annotated the corresponding TFs by assaying the enrichment with PWYs and GO terms of their highly similar genes (Figure S6a). In total, we found 23,796 TF-PWY and 7,722 TF-GO significant associations (FDR ≤ 0.1, Fisher’s Exact) (Figure 2a) (Table S8), which on average captured ~7 PWYs and ~8 GO terms per TF (Figure 2b). Combining both assays, we annotated 2,910 different TFs, out of which 1,030 showed enrichment with both PWYs and GO terms (Figue S6c). To note, these 1,030 TFs belong to 82 different families (including co-regulators) representing - on average - 34% of the total proteins annotated in the corresponding families (Figue S6d). This highlights the potential of the method to annotate TFs with unobserved interactions, e.g., without a previously published ChIP-seq.

Comparing all three methods, the *network-based* approach enabled us to identify the largest total number of TF-PWYs, and the lowest total number of TF-GOs associations (Figure 2a). Additionally, this approach had the lowest average of PWYs and GO terms per TF (Figure 2b). Unexpectedly, *network-based* and *common target* methods predicted a similar number of PWYs per TFs, which contrasts with the significantly lower number of GOs between *network-based* and the other two methods (Figure 2b). Importantly, the number of TF annotated by the *network-based* method is >2.5 times larger than the other two methods (Figure 2.c, left panel). Thus, by using the *network-based* approach, we functionally annotated 2,917 TFs.

### Evaluation of functional predictions

TF perturbation experiments enable the understanding of the TF regulatory landscape by unraveling the direct and indirect effects of expression changes induced by the expression variation of the corresponding TFs. Here, we used 21 previously published knockout assays associated with 13 different TFs (Table S9) (Zhou et al., 2020; Ellison et al., 2023) to assay the accuracy of each of the three methods by two independent strategies. Specifically, we questioned the overlap between predicted and observed PWYs/GO terms within DEGs for the corresponding knockouts. In parallel, we also tested the gene set enrichment analysis (GSEA) of the predicted PWYs/GO terms within the corresponding TF knockouts (Subramanian et al., 2005) (See Methods). Overall, predicted PWYs from the networks - without distinction of the methods - showed poor overlapping with PWYs observed in the knockout assays, as well as low recovery of PWY significantly enriched within DEGs as estimated by the GSEA (Figure S7). Conversely, comparisons between predicted and observed GO analyses showed similarities [measured by the GO semantic similarity (GSS)] different than the expected by chance (P-value ≤ 0.05) (Figure S8). Overall, the GO terms from knockouts and the GO terms predicted by *network-based* and *common function* are significantly more similar than the *common targets* predictions (higher GSS values, P-value ≤ 0.05, Wilcoxon test) (Figure 2d). When a prediction was available, the *network-based* method recovered the GO terms with the highest GSS values among all the methods (Figure 2d, TB1 and FEA4 results). Additionally, we observed that seven knockouts lacked predictions from *network-based* methods, while eight others had predictions only with *network-based* methods (Figure 2d, e). This variability in predictions can be partly attributed to the low number of target genes (when the prediction is absent; Figure S9a, TFs with Z-score ≤ 0) and the absence of data in at least one of the four layers (i.e., GRN, eGRN, CEN, and GAN) (when the *network-based* method is the only one making the prediction, Figure S9b). Consistently with the GSS analysis, the GSEA results indicate that GO terms recovered with the *network-based* and the *common function* are more consistently identified across the different knockouts (Figure 2e). Thus, together our results indicate that *network-based* predictions are robust to presence-absence variation in the data across layers. It is, however, susceptible to the number of targets by TF. By extension, our findings also indicate that *common targets* and *function* predictions are more sensitive to the absence of data in at least one of the layers.

Combining all GSEA results, we found that, on average, only 25% of total GO predictions show significant GSEA scores (P-value ≤ 0.05), denoting a low recovering rate of GO terms (Figure 2e). We used the tissue specific expression of TFs as a proxy to explain better the relationship between the low fraction of GO recovered by GSEA and the tissue/condition/genotype variation among the corresponding TFs. Including all the TFs for which we obtained at least one PWY/GO term prediction and using the Tau index as a metric (Kryuchkova-Mostacci and Robinson-Rechavi, 2017), we find a bi-modal expression distribution with ~55% of the TFs trending into a sample-specific expression fashion (Figure S9c, Tau ≥ 0.65). Interestingly, only four out of the 13 TFs tested in the knockout analyses are expressed in a tissue-specific fashion, e.i, these TFs tend to be expressed in a few conditions (P-value ≤ 0.05) (Figure S9d, labeled in green). The top four included RAMOSA1 (RA1) (Tau 0.99) and TEOSINTE BRANCHED1 (TB1) (Tau 0.96), which also are the top two TFs with the largest fraction of GO term supported by the GSEA (Figure 2e). Hence, the results support the notion that part of the low fraction of overhauling between the GO terms predicted compared with observed annotation from the knockout assaults can be attributed to the differences in conditions used on the knockout and prediction analyses.

We also evaluated the identification of GO terms from random networks to determine which method recovered the lowest fraction of false positives (Supplemental data 2 and Figure S10). Overall, we observed a high degree of overlap between the prediction observed in *the common target* and the *common function* methods, suggesting the two methods were equally noisy. Consequently, *network-based* method predictions always lower the number and have more differences in GO terms compared to the observed GO terms from our predictions (Figure S10). In conclusion, the *network-based* method is a superior approach within the given context of these datasets. Consequently, we exclusively relied on *network-based* predictions for subsequent analyses.

### Prioritization of regulators by biological process

**Figure 3.**
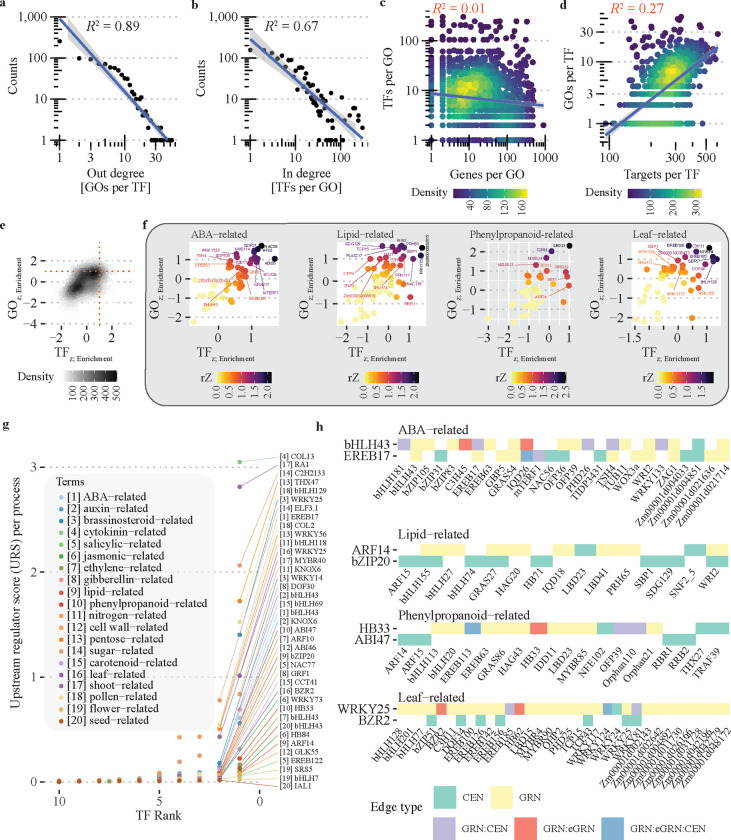
Prioritization of regulators by biological process using network-based prediction. Out degree (**a**) and in degree (**b**) distributions of the TF-GO term predictions obtained from the network-based integration analysis. **c** and **d**, scatter plots indicating the frequency - as density - of the number of TFs by GO terms (in degree) and GOs per TF (out degree) as a function of the number gene annotated per GO term (**c**) and target genes per TF (**d**), respectively. **e**, Scatter plot indicating the frequency - as density - of the TF-GO term enrichment scores scaled, which allows to rank GOs highly enriched with specific TF (GOz) and TFs highly enriched with specific GO term (TFz). The enriched was calculated only with TF-GO term associations already predicted in previous analysis. Dotted line orange indicates TF-GO term associations with enrichment score a standard deviation over the observed average for the corresponding TF and GO term (Z-score of enrichment ≥ 1 for both GO term and TF). **f**. Scatter plot with reciprocal Z score (rZ) of four different biological process mapped into the GO and TF scaled score coordinates as presented in **e**; GO terms were grouped as follow: ABA-related (GO:0009737, GO:0009738, GO:0009688, and GO:0009788), Lipid-related (GO:0031408, GO:0006099, GO:0006635, GO:0019915, GO:0006629, GO:0019375, GO:0044255, GO:0016042, GO:0051790, GO:0008610, GO:0009062, and GO:0045332), phenylpropanoid-related (GO:0009963, GO:0009698, GO:2000762, and GO:0009699), and leaf-related (GO:0009965, GO:0048366, GO:0010305, GO:0010150). TF name/gene id labels are included for TFs with rZ ≥ 0.5. **g**. Scatter plot with TF ranked their upstream regulator score (URS) by biological process. TF name labels are included for TFs with rank ≤ 2. Square brackets indicate an arbitrary biological process index which matches with the number in square brackets of the corresponding TF names. All URS scores are calculated based on the original GRN, eGRN, CEN and GAN networks. **h**. Heatmap with top two TFs (y axes) from the URS analysis (**g**) for the four biological processes presented in **f**. X axes indicate the corresponding TF targets. Colors indicating the network(s) source of the corresponding interactions.

The network-based method detected approximately 7.7K TF-GO associations, encompassing 1,036 TFs and 2,219 GO terms (Figure 2a, c & Figure S6b, c). For ease of TF comparison, we retained associations involving GO terms within the biological process (BP) category and having fewer than 800 associated genes (when a more specific GO term association was present). Additionally, to minimize GO term redundancy, we mapped GO terms with less than 50 genes to their nearest GO term parent. After applying the filters, we continued with 4,337 TF-GO associations, comprising 902 TFs and 559 GO terms. The distribution of TF-GO associations obtained holds a scale-free distribution (Figure 3a, b). Typically, highly interconnected GO terms and TFs suggest a greater number of annotated and targeted genes, respectively. Nevertheless, we did not discover any evidence linking the gene count per GO term or the target gene count per TF to their respective degrees (Figure 3c, d). Therefore, these analyses highlight GO terms whose regulation may depend on multiple TFs, and TFs that may contribute to regulating several biological processes.

From the perspective of gene regulation, when multiple TFs are associated with multiple GO terms, it suggests that the regulatory impact of a TF on a particular GO term is influenced by the presence of other TFs. To assess the contribution of individual TFs to their respective GO terms, we calculated a scaled enrichment score (Z-score of the enrichment) for each TF and GO term (See Methods). Utilizing the scores as indicators to assess the strength of individual TF-GO associations relative to all other TF and associated GO terms, we observed that only 3.4% (151/4,337) of the TF-GO associations exhibit high enrichment scores (Z-score ≥ 1) (Figure 3e, top right corner), indicating that only a reduced fraction of the TFs and GOs analyzed have strong enriched scores for the corresponding association. This implies that most of the analyzed TFs/GO terms have multiple associations of comparable significance. We combined both Z-scores (per TF and GO term) into a reciprocated Z-score (rZ, See Methods) to rank TFs by GO term using a single metric. We evaluated the ranking after grouping GO terms into specific biological processes (Figure 3f, and Figure S13). We highlight here, 46 abscisic acid (ABA) metabolism-, 62 lipid metabolism-, 47 phenylpropanoid metabolism-, and 50 leaf development-related TF-GO associations that were targeted by 44, 55, 47, and 50 different TFs, respectively (Figure 3f). Using the rZ score as filter (rZ ≥ 0.5), we narrowed down the list to 25, 27, 15, and 19 TF candidates to control the corresponding processes (Figure 3f, dots with name label included). Some examples included the top two TFs ABA metabolism related, NAC56 and WRINKLED2 (WRI2). Additionally, WRI2 was also on the top three of the TFs related to lipid-related metabolism (Figure 3f, second panel). Finally, five (WOX9a, OFP39, Zm00001d024353, EREB149, and LBD24) out of the initial 47 phenylpropanoid-related TFs were previously identified as maize regulators of phenolic-related genes by yeast-one hybrid assays (Y1H) (Yang et al., 2017). Altogether, this highlights the biological relevance of the associations predicted by this analysis.

Apart from controlling specific enzymatic or signaling-related genes, TFs can also regulate biological processes by targeting other TFs. This leads to the formation of regulatory circuits with multiple hierarchical levels and network motifs. To identify TFs that play a higher-level role in controlling a biological process, we calculated the ratio of TFs targeted by other TFs within specific GO terms to the total number of TFs targeted by the corresponding TF. Given that we searched for TF associated with a common function, this ratio represents the weighted proportion of feed-forward loops associated at the level of biological process compared to the overall TF targets of the corresponding TFs. This measure is referred to as the upstream regulator score (URS). We calculated the URS for the twenty different processes, including eight hormone-, seven metabolic-, and five developmental-related processes (Figure 3g). Cytokinin- and shoot-related GO terms were the top two processes with the highest score, with COL13 and RA1 as their top regulators, respectively (Figure 3g). To note, COL13 was previously associated with carbon metabolism (Tu et al., 2020), and is also differentially expressed in the *indeterminate1* (*1d1*) loss-of-function mutant (Minow et al., 2018). ID1 is a maize regulator of autonomous floral induction (Colasanti et al., 1998). Thus, our results suggest a role of COL13 in the connection among cytokinin, carbon metabolism, and flowering; mechanistic association previously reported in other plant systems (Bartrina et al., 2011; Wahl et al., 2013). Similarly, RA1 was predicted as the top upstream regulator of shoot-related processes, and RA1 itself was linked with shoot system development (Figure 3g, process number 17), both of them functions previously associated with RA1 (Eveland et al., 2014). To further understand the regulatory landscape of the four processes described previously (Figure 3f), we selected the top two predictions - URS score - for each process and traced their TF targets back to the original networks (*i.e*., GRN, eGRN, CEN, and GAN) (Figure 3h). Specifically, we looked for regulators directly upstream of any of the top TFs as predicted by the reciprocal Z-score (rZ) analysis (Figure 3f). Without exception, we found at least an upstream regulator directly targeting (GRN network) at least one of the top three TFs from the rZ analysis, *i.e*, a top regulator of the corresponding biological process (Figure 3h). To highlight an example within the ABA-related process network, EREB17 targeted NAC56 and WRI2 (tops TFs in rZ analysis), and bHLH43 (URS top one) targeted WRI2 and EREB17 (Figure 3f, h, first panel). This configuration forms a feed-forward loop with bHLH43 on top (*i.e*. bHLH43 targets EREB17 and WRI2, and EREB17 targets WRI2). Within the lipid-related network, ARF14 targeted WRI2 and PRH65 (top two and three by rZ score) (Figure 3f, h, second panel), as so did HB33 target LBD23 (top in rZ) in the phenylpropanoid-related network (Figure 3f, h, third panel). Finally, WRKY25 (top USR) targeted the MYBR4 and EREB126 (top two TFs in rZ), as well as BZR2 (top two in URS analysis) on the leaf-related network. Thus, our results predicted specific regulatory interactions for further experimental validation.

### Mapping TF-GO process into specific conditions

TFs generally work in a tissue/condition-specific manner. Thus, to gain a better understanding of the regulatory condition that most closely captures the functional space of the corresponding TFs, we performed a gene set enrichment analysis (GSEA) with the 4,337 TF-GO associations predicted to map these associations to specific developmental and environmental conditions. Specifically, we used the expression data utilized previously in the constructions of the 45 co-expression networks (Zhou et al., 2020), as well as, additional six expression datasets of maize plants subjected to heat and cold stress (See Methods) (Zhou et al., 2022). Unlike a typical GSEA, we employed the Pearson correlation coefficient (PCC) as the gene ranking metric, as we described previously (Gomez-Cano et al., 2022). It is worth noting that this analysis does not discover new TF-GO associations. Instead, this GSEA ranks each of the previously described TF-GO term associations in the context of the expression datasets tested (referred here as co-expression networks). Consequently, we first determined the percentage of the co-expression networks where the corresponding TF showed a significant gene-set enrichment (FDR ≤ 0.1) with at least one of their associated GO terms. On average, 76% of the tested TF-GO terms exhibit significant co-expression (FDR ≤ 0.1) in at least one network (Figure S14a). This suggested that most of the TF-GO term associations tested may be mapped to a particular biological condition. Moreover, we calculated the average percentage of significantly co-expressed TF-GO term associations in each co-expression network per TF. This helped us understand the functional diversity of each TF. In general, each TF had 53% of GO terms that showed significant enrichment in each co-expression network (Figure S14b). It is worth noting that TFs with the lowest and highest average percentages of significantly co-expressed GO terms also possessed the lowest number of associated GO terms (Figure S14c). Thus, this uncovers TFs with limited associations and completely functional space, i.e., TFs associated with few GOs and co-expressed with them in either a few conditions or in almost all of them.

**Figure 4.**
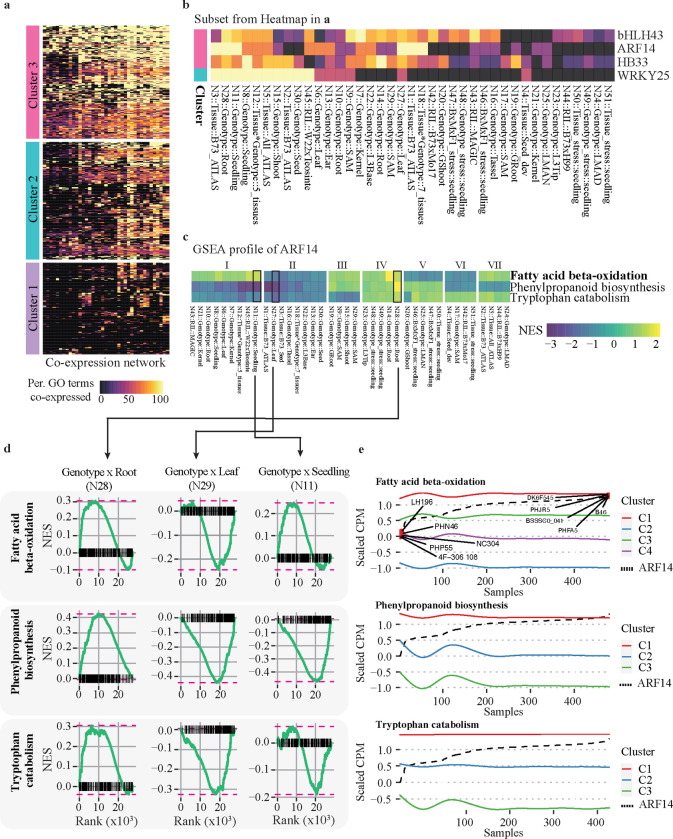
Mapping to TF-GO terms associations to specific conditions. Heatmap showing the total (**a**) and a subset of four TFs (**b**), indicating the percentage of GO terms significantly enriched in the GSEA analysis for each TF across the respective co-expression networks tested. **c**. Heatmap displays the normalized sentiment score (NES) of ARF14 and its three associated GO terms across 39 networks where its GSEA was assessed. GSEA results are not shown in the remaining 12 networks due to its absence of ARF14’s expression or the lack of statistically significant (FDR > 0.1) in all three GO terms. **d**. GSEA profile from three networks in **c**, displaying the actual accumulation of gene sets along the ranking established based on ARF14’s co-expression with the whole maize genome. **e**. Locally weighted scatterplot smoothing (LOESS) line plot with the scale CPMs of the gene set in **d** across different samples within the respective networks. Genes were clustered after normalizing and scaling their expression values. The dashed line represents the scaled expression of ARF14, with samples on the x-axis organized according to ARF14 expression levels.

Further analysis of the percentage of co-expressed GO terms per TF allowed us to identify three clusters of TFs (Figure 4a). These clusters consisted of 291 TFs in cluster 1, which captured TFs with the highest average percentage (69%) of GO terms co-expressed by network, while cluster 3 includes 235 TFs with the lowest percentage (45%). Cluster 2, on the other hand, encompasses 303 TFs where approximately half (50%) of their GO terms are significantly co-expressed in at least one of the tested networks (Figure S14d). Additionally, these clusters also differ in different numbers of co-expression networks by TF. On average, cluster 1 TFs have significant associations with up to 29 different networks, while cluster 2 and 3 showed signals in 18.4 and 6.2 different networks out of the 51 total networks analyzed, respectively (Figure S14e). Comparison of the GO terms by TF indicates that TFs in cluster 3 tend to have a larger number of GO terms. However, we did not find significant differences between cluster 1 and 3 (Figure S14f). The latter suggests that a large fraction of the TF-GO terms association in cluster 3 fail to provide relevant biological information, at least based on the condition tested here (Figure S14d). However, this does not explain the low number of co-expressed GO terms by networks (Figure S14e). Therefore, we concluded that the set of TFs in Cluster 3 serves multiple functions that operate conditionally. This contrasts with TFs in Clusters 1 and 2, which are associated with a smaller number of GO terms but have a potentially broader scope of influence.

To explore the biological significance of the TF-GO mapping strategy, we focused on bHLH43, ARF14, HB33, and WRKY25, previously described as upstream regulators of different biological processes. Specifically abscisic acid response-, lipids metabolism-, phenylpropanoid metabolism-, and leaf development-related processes, respectively (Figure 3g–h). We propose that they might have regulatory roles across a wide range of biological conditions. Our analysis revealed that bHLH43, ARF14, HB33, belonged to cluster 1 (Figure 4b). WRKY25 mapped to cluster 2, which has significant TF-GO terms association in almost all the co-expression networks tested (Figure 4b). We delved further into the GSEA results to gain a deeper understanding of the corresponding TFs regulation dynamics. To identify potential activation or repression activities, we relied on the normalized gene set enrichment score (NES). A positive NES suggests enrichment in positive correlations, while a negative NES suggests enrichment in negative correlations. This is the assumption given that we used PCC as the ranking metric on the GSEA analysis (See Methods). We observed that multiple GO terms per TF show similar enrichment patterns across different networks for the four TFs. For example, bHLH43, upstream regulator of the response to abscisic acid (GO:0009793), exhibits a similar enrichment pattern in the regulation of multicellular organismal development (GO:2000026) and embryo development leading to seed dormancy (GO:0009793) (Figure S15a). Similarly, HB33, predicted as regulator of phenylpropanoids (GO:2000762), shows a similar enriched pattern in the regulation of hydrogen peroxide metabolic processes (GO:0010310) and regulation of plant-type hypersensitive response (GO:0010363) (Figure S15b). Also, WRKY25, which is associated with only two GO functions - leaf morphogenesis (GO:0009965) and brassinosteroid biosynthetic process (GO:0016132) (Figure S15c) - shown similar enrichment NES values (PCC > 0.7) in 20/24 networks, suggesting a potentially significant functional connection between leaf morphology and the regulation of brassinosteroid biosynthesis. Finally, ARF14, predicted as an upstream regulator of lipid-related processes (specifically fatty acid beta-oxidation, GO:0006635), also has an enrichment profile similar with tryptophan catabolic process (GO:0006569) and phenylpropanoid biosynthetic process (GO:0009699). All three metabolism pathways are known for their interdependency because fatty acids metabolism may influence the production of several phenylpropanoids and because phenylpropanoids and tryptophan utilize similar precursors from the shikimate pathway (Maeda and Dudareva, 2012; Perez de Souza et al., 2020). It is worth noting, the direction of NES values also indicate that this functional association is conditions-specific (Figure 4c).

We selected three networks to understand the relationship between ARF14 and its associated pathway. Specifically, we selected a network in which three pathways showed positive NES score (N28, genotype by root variations), negative NES score (N29, genotype by leaf variations), and combined effects (N11, genotype by seedling variations) (Figure 4d). Then, we plotted the GSEA profiles which showed the gene set accumulation profile along the gene set rank. In all three cases, the GSEA profile validated the dynamics suggested by the NES score in terms of the gene set rank set pattern. However, we noticed that the highest and lowest positive and negative NES peaks occurred around the 5,000 and 20,000 gene ranks, respectively (Figure 4d, marked with dashed pink lines). This indicated that a large number of genes related to the corresponding pathways are not directly correlated with the corresponding TF. We also observed a double weak peak in the case of tryptophan catabolism and fatty acid beta-oxidation in seedlings (Figure 4d, right panel - network N11), suggesting that ARF14 may have a positive and negative expression effects on the corresponding pathway even within the same network. To test our hypothesis, we specifically examined network 11, which displayed a variable NES sign across all three pathways associated with ARF14. We grouped the genes corresponding to each pathway based on their expression profiles. Then, we plotted the expression of ARF14 across the relevant samples (Figure 4e). As anticipated, we found a subset of genes that exhibited expression changes not related to ARF14 (labeled as C1 in Figure 4e). We also observed that there is a group of maize lines in which an increase in ARF14 expression aligns with increased expression of the phenylpropanoid pathway (positive NES peak in Figure 4d, N11 middle panel), which also exhibited an increase in ARF14 expression (Figure 4d, Samples 50 to 150). This observation supports the idea of different effects even within the same pathway. Finally, since this network was constructed based on genotype variations, we can identify the specific lines where ARF14 has its lowest (left labeled dots in Figure 4e, top panel) and highest (right labeled dots in Figure 4e, top panel) expression levels. This corresponds to lines where manipulating ARF14 would be expected to have the greatest and lowest impact on the associated target pathways.

### Topological properties predict TF paralogs redundancy.

**Figure 5.**
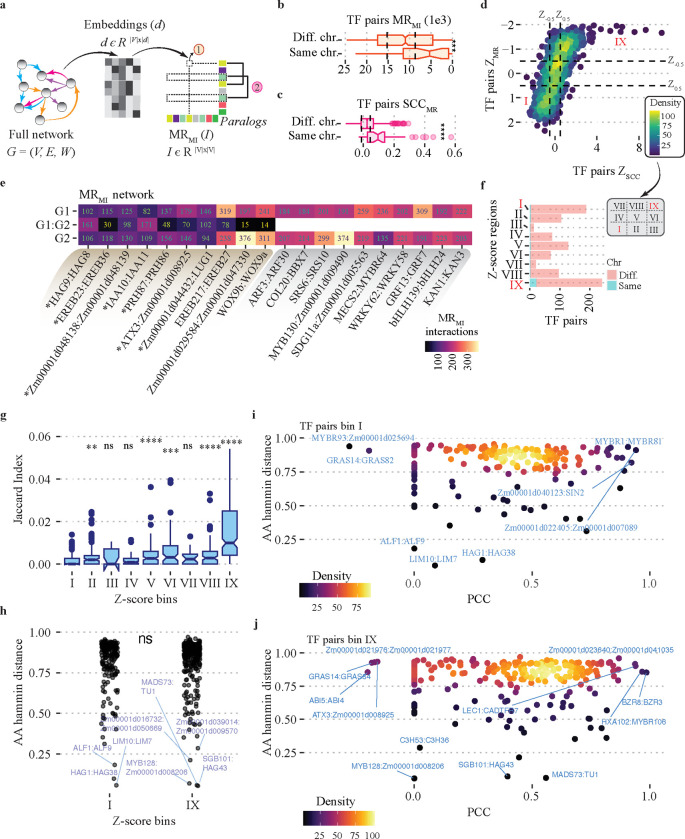
Network embedding as predictor of TF paralogs with functional variation. **a**. Diagram illustrating the key stages of comparing TF paralogs through embedding similarities. **b** and **c**. Box plots displaying the MRMI of TF pairs (**b**) and the Spearman correlations (SCC) of the observed MRMI profiles (**c**) derived from the embedding. d. Combined scaled scores of the MRMI and SCC for TF pairs. Black dashed lines with Z-scores of −0.5 and 0.5 indicate values below and above the average observed standard deviation. **e**. Heatmap indicating the total number of associated genes for the top ten TFs, on the top right corner and the bottom left corner TF pair (**d**). G1 and G2 represent the number of unique genes associated with the first and second TFs in the corresponding pair. G1:G2 indicates the common associations between the corresponding pair. F. Bar plot indicating the total number of TF pairs by bin. Bins are indicated on the interval box, which is a map of the zones in the plot in (**d**). **g**. Box plot with Jaccard index (as an approximation of common associated genes) by TF pair by bin (as presented in **f**). **h**. Jitter plot displaying amino acid (AA) differences (Hamming distance) between TF pairs in bins I and IX. **i** and **j**, Jitter density of points representing AA Hamming distance and co-expression (measured as PCC) for TF pairs in bin I (**i**) and IX (**j**). Asterisks indicate P-value significance (*: p ≤ 0.05, **: 633 p ≤ 0.01, ***: p ≤ 0.001, ****: p ≤ 0.0001, two-sided t-test). “TFm” denotes multiple mutant lines for the same TF.

Although substantial efforts have been made to comprehend and anticipate the functional redundancy between maize paralogs in subgenomes (Schnable et al., 2011; Li et al., 2016; Kono et al., 2018; Han et al., 2023), it remains challenging. We anticipate that if a pair of paralogs exhibit functional redundancy, these differences may manifest in their topological properties, *i.e.*, functional redundant paralogs would display similar properties indicating a comparable network arrangement. To assess the similarity between paralogs, we generated a distance matrix from the embeddings using the mutual rank (MR) of mutual information as metric (Figure 5a). Next, we mapped TF paralogs (Schnable, 2019) and analyzed their MR (Figure 5a, organ circle) and the similarity of their MR profiles (Figure 5a, pink circle) with all the genes in the embeddings matrix. We used these metrics as a proxy for understanding the similarity of their embeddings and the similarity of their resemblance with other TFs examined, respectively. We also differentiated between paralogs located on the same chromosome, serving as a reference for pre-speciation tandem duplicates. In total, we tested 932 TF pairs, and regardless of the metric used, TF paralogs situated on the same chromosome demonstrated greater similarity compared to TF paralogs on different chromosomes (Figure 5b, c). We combined both scaled metrics to identify highly similar TF pairs (Figure 5d). As expected, both metrics were correlated (PCC 0.68), yet they effectively served the purpose of identifying TF paralog pairs that were highly similar. We tallied the number of interactions after the embedding integrations (Figure S6a, b) for the top ten TF pairs that were most and least similar (Figure 5e). The top ten most similar TF pairs have several common interactions (Figure 5e, TF pairs highlighted in light brown), contrary to those observed within the top least similar, which have none (Figure 5e, TF pairs highlighted in gray). Additionally, seven TF pairs mapped to the same chromosome out of the top ten most similar TF pairs (Figure 5e, TF pairs with asterisk). This last supports our prediction of similarity in topological properties as a potential predictor of paralogs functional redundancy. To gain a better understanding of the similarities in the embedding within tandem duplicates, we organized all TF pairs into nine categories using scaled similarity metrics (Figure 5d, areas defined by the dashed black lines). These categories range from the least similar TF pairs in the first category (I) (Figure 4d, left bottom corner) to the most similar pairs in the last category (IX) (Figure 4f, right top corner). We then compared the number of paralogs that are tandem duplicates and those that are not, which confirmed the observation from the top ten TFs (Figure 5e), exemplified by bin IX containing 5–7 times more tandem duplicates than the other bins (Figure 5f). We also quantified shared interactions using the Jaccard index. By considering bin I as a reference, we detected significant differences (p < 0.05, two-sided t-test) across five distinct bins (Figure 5g), primarily categorized based on the scaled correlation (Z_SCC_) between TF pairs (Figure 5d, x axes). Furthermore, bin IX exhibited the utmost values, validating the predictive capacity of embedding similarities for functional redundancy in TF paralogs.

We speculate that a low similarity in topological properties indicates functional divergence. On the other hand, a high similarity suggests conservation of function. Therefore, we would anticipate observing similar patterns of variation either in the protein sequence or in its regulation, along with the inherent similarities between corresponding pairs of paralogs. Specifically, we expect to see greater differences in either the protein sequence or the expression of paralogs in bin I compared to the other bins. To investigate if this is the case, we focused only on TF pairs from bins I and IX, which represent the most different pairs. We calculated the Hamming distance of the amino acid sequences, which measures the number of changes in amino acids between the corresponding paralog pairs (Cocco et al., 2018; Facco et al., 2019). Additionally, we analyzed TF paralogs co-expression as an indicator of variation or conservation at the *cis*-regulatory level (Zheng et al., 2011; Ruan et al., 2011; Kulkarni and Vandepoele, 2019). We did not observe any differences in the Hamming distance between TF paralogs highly similar or dissimilar at the topological level, as evidenced by TF pairs highly conserved (low Hamming distance) in both groups of TFs (Figure 5h). However, TF pairs in bin I showed slightly lower average co-expression (PCC = 0.4) compared to those observed for TFs in bin IX (PCC = 0.5). Yet, the mapping of the co-expression along the Hamming distance for the corresponding TF pairs, allowed us to differentiate TF paralogs that may be undergoing neofunctionalization/subfunctionalization due to variations in their protein sequences or its expression profiles. A striking example of the former is observed in MYBR1 and MYBR81, which have large numbers of changes in their sequences (Hamming distance close to 1), distinct embedding profiles (bin I), and yet display high co-expression (PCC > 0.9) (Figure 5i). In contrast, HAG1 and HAG38, as well as GRAS14 and GRAS82, which also belong to bin I, shown similar peptide sequences (Hamming distance close to 0) and different expression (PCC < 0.3), suggesting variation at the regulation level (Figure 5i). Additionally, within the groups of TFs sharing similar embedding profiles (bin IX), we identified TFs exhibiting high sequence conservation (Hamming distance close to 0) and similar expression, implying a significant degree of redundancy (e.g., MADS73 and TU1) (Figure 5j). Continuing with TFs within bin IX, we also find TFs with low co-expression but high conservation (Hamming distance close to 0, e.g., C3H53 and C3H36), as well as TFs with poor sequence conservation (hamming distance close to 1, ABI5 and ABI4), indicating differences in its regulation (Figure 3.4j). Taken together, the combination of embedding similarity, protein amino acid similarities, and co-expression enables the identification of TFs that are either potential variable or redundant, a key observation for understanding functional redundancy.

## DISCUSSION

Cells utilize complex networks of molecules to integrate and synergistically regulate their activities. The rapid generation of multi-omic genomic data in plants has led to growing interest for stabilizing integration strategies (Liu et al., 2016; Walley et al., 2016; Wen et al., 2016; Jin et al., 2017; de Abreu E Lima et al., 2018; Lee et al., 2019; Schaefer et al., 2018; Wen et al., 2018). In this study, we analyzed distinct data types (PDI, expression, and SNP) and constructed four different molecular networks (layers). Considering the inherent challenge posed by the variations in each respective network, we examined three distinct integration methods and employed two different strategies to functionally annotate TFs (Figure 1). Overall, our findings indicate that the integration of multiple layers based on *common targets* and *functions*, although more intuitive, are less accurate in recovering observed GO terms in knockouts (Figure 2d, e). Instead, it frequently yields results that can be readily attributed to chance, as demonstrated by the number of times that a GO term may be retrieved from random networks (Figure S10a). Focusing on the *network-based* integration to prioritize transcriptional regulators associated with specific biological processes, we gained valuable insights into potential regulatory mechanisms underlying maize metabolism - as well as developmental-related processes. Using GSEA, we mapped TF-process associations to specific biological conditions. Thus, we presented specific conditions in which the corresponding TF may play a major regulatory role in the corresponding biological process. Finally, we show how the generations of embedding after integrating the multiple networks allow for highlighting differences in TF paralogs, representing an alternative strategy to uncover the functional diversification of paralogs in maize. Altogether, paving the way for the multi-omic integration of similar datasets in other plant systems. Similarly, we argue that our findings will guide the designing of specific experiments aimed at crop improvement, metabolic engineering, and basic gene regulation understanding of the corresponding processes.

Worthnothy, the *common targets* and *common function* strategies predict significantly more similar GO terms in random networks than the *network-based* method (Figure S10a, b). From a technical perspective, this also suggests that the initial set of interactions may contain a significant number of false positives, which explains why random networks can recapitulate such a large number of these functional associations. Furthermore, it also indicates that even when a TF-target interaction is highly reliable (due to its presence in multiple layers), it alone is insufficient to provide an accurate representation of the biological landscape associated with the corresponding TF (Figure 2d). Interestingly, unlike the first two methods, the *network-based* method proved to be more resilient to the presence of false positives, as indicated by the results from the random networks (Figure S10). Our contention is that this resilience is rooted in the inherent nature of the embedding generation process, as it is highly improbable to observe similar wiring patterns across layers, despite the expected presence of false positives within each respective layer. Additionally, of equal significance, the *network-based* approach facilitated predictions for a considerably larger number of TFs (Figure 2c), thereby influencing the design of future experiments aimed at uncovering and validating specific TF functions in maize.

Combining all the TF-function predictions made by our *network-based* integration, we find a network-like structure independent of the number of genes by GO term or targets by TF (Figure 3a–d). Using a scaled enrichment score for each TF and GO term, we showed that only ~3% of our prediction had a single TF as the primary regulator of the corresponding GO term, consistent with the notion that the vast majority of TFs contribute to the regulation of multiple functions, and that the regulation of a biological process requires the involvement of multiple TFs (Tu et al., 2020; Ledezma-Tejeida et al., 2019; Tang et al., 2021; Yang et al., 2017). This last is a distinctive feature of GRNs, the regulatory repertoire of TFs is leveraged through interactions with other TFs (Reményi et al., 2004; Brkljacic and Grotewold, 2017). This phenomenon has been previously observed in maize, both at the level of TF-target genes (Yang et al., 2017; Tu et al., 2020) and in the organization *cis*-regulatory elements across cell types (Marand et al., 2021), supporting our results. Additionally, we prioritized TFs by biological process combining scaled scores from both TF and GO terms and built two tier regulatory models for specific biological processes. We showed the presence of a feed-forward motif within our results, which is known as a mechanism for reinforcement of regulatory signals (Alon, 2007). Thus, our findings extend the concept to a broader framework, encompassing biological processes, and have significant implications for future biotechnological applications, such as the targeted modification of specific metabolic processes.

TFs generally work in a tissues/condition-specific manner (Sonawane et al., 2017). Here, we mapped our TF-GO function prediction into 51 different datasets encompassing around 7,000 different RNA-seq datasets (Zhou et al., 2020, 2022) using GSEA and TF-gene correlations as ranking metric (Gomez-Cano et al., 2022). Our analysis revealed different types of TFs based on the number of conditions in which we observed significant enrichment with the corresponding GO terms (see Figure 4a). Most TFs, including those in clusters 1 and 2 (Figure 4a), regulate multiple GO terms across various conditions. However, a smaller percentage of TFs are involved in regulating their corresponding GO terms in a limited number of conditions (cluster 3, Figure 4a). This emphasizes the existence of a condition-specific complex regulatory landscape. Additionally, since multiple TFs can contribute to the same function (Figure 2f), the results suggest that the specific behavior in each condition is established by the combined action of multiple TFs instead of individual TFs, as suggested by previous studies (Sonawane et al., 2017).

We focused on four different TF examples that our analysis suggested as potentially significant regulators of various metabolic and developmental processes (Figure 3h). Our GSEA enrichment results reinforce this observation, as they show the remarkable presence of significant association in numerous conditions (Figure 4b). We highlighted ARF14, TF that is known to play a role in auxin response factors (Cancé et al., 2022). According to our analysis, ARF14 is identified as a regulator of three metabolic pathways, including tryptophan catabolism. Since tryptophan serves as an important precursor for indole-3-acetic acid (IAA), the primary auxin in plants (Zhao, 2010), this finding highlights a potential functional connection between hormonal and metabolic control.

Furthermore, our GSEA analysis suggests a co-expression coordination between each pathway and ARF14, but this coordination is specific to particular conditions, further supporting the concept of interaction between metabolic pathways (Vanstraelen and Benkova, 2012; Derksen et al., 2013; Munné-Bosch and Müller, 2013; Pacifici et al., 2015; Sawicki et al., 2015). Specifically, the NES value suggests that in seedling, leaf, and kernel, ARF14 may have a slightly positive association with lipid-related processes (Figure 4c, cluster I). Yet, in similar conditions, it has either no association or a slightly negative association with phenylpropanoid synthesis and tryptophan catabolism (Figure 4c, cluster I). Similarly, when only B73 data is included (ATLAS datasets), seed, or any reproductive organ, ARF14 loses its influence on two pathways (Figure 4c, cluster VI) or all three pathways (Figure 4c, cluster II, VII). Contrastly, data from shoot apical meristem and mature shoot suggest that ARF14 could promote phenylpropanoid biosynthesis and induce fatty acid beta oxidation (Figure 4c, cluster III) or the breakdown of tryptophan (Figure 4c, cluster V) at the same time. Finally, when the networks include root, SAM, or seedling under stress, the associations of ARF14 with all three networks become positively enriched (Figure 4c, cluster V). Altogether, our results indicate that there is a condition-specific coordination (defined here as a similar NES score) between these pathways.

The understanding of functional redundancy between maize subgenomes remains far from complete (Schnable et al., 2011; Li et al., 2016; Kono et al., 2018; Han et al., 2023). In this study, we used the similarity of embeddings generated after molecular network integration to identify TFs functionally redundant. Our findings show that TF paralogs with more similar embeddings have more common targets and are often found as duplicates in tandem (Figure 5e–g). While we did not find significant differences in the variation of amino acid sequences between TF paralogs with low or high similarity in embeddings space (Figure 5h), we noticed slightly higher co-expression similarities between TF paralogs with closer topological similarities. Thus, it suggests a relationship between the regulation and embedding similarities of the corresponding TF paralogous. This agrees with previous reports that highlights changes in regulatory regions as major drivers of phenotypic variation (Schmitz et al., 2022; Marand et al., 2023). Although we can’t fully explain the reasons behind the similarity in network connections between TF paralogs, it is important to note that by integrating information about proteins sequence, expression, and embedding similarities, we can identify specific candidates for functional neofunctionalization/subfunctionalization and redundancy (Figure 5i, j). These observations need to be tested through experimental validation.

In summary, our findings demonstrated that integrating all multi-omic datasets through embedding identification, followed by clustering, allows us to identify genes with similar wiring on the fully integrated network. It is worth mentioning that the embedding similarities create an association network themselves, encompassing gene-gene associations for over 24K genes. Here, we used this strategy to annotate TFs. However, this also defined a conceptual framework for expanding this annotation strategy to the other types of genes, e.g., the annotation or definition of metabolic pathway based on the topological embedding similarities.

## METHODS

### Genetic markers

A set of 304 diverse inbred lines with publicly available SNP and gene expression information were included in our eQTL analysis (Bukowski et al., 2018; Kremling et al., 2018; Mazaheri et al., 2019). Given that the previously published SNP marker data from whole genome sequencing and RNA-seq were mapped to the same B73 maize reference genome (AGPv4) (Jiao et al., 2017), we performed a simple concatenation of the datasets using bcftools (v1.7) (Danecek et al., 2021). In the case that an overlap was observed between the two datasets, the RNA-seq marker was preferentially kept. The expression datasets capture variation both at the genotypic and tissue level.

### RNA-seq and co-expression analyses

All the RNA-seq and co-expression datasets utilized here were previously published (Zhou et al., 2020), except for co-expression network 46, which was constructed using the 304 inbred lines analyzed for genetic markers (referred to as n304). Specifically, we gathered pre-mapped CPM values for the respective inbred lines and employed the exact strategy outlined by Zhou et al. (2020) to construct the corresponding co-expression networks, ensuring comparability among all 46 networks. All co-expression networks were based on RandomForestRegressor and using the top 100K association by TF.

### eQTL identification and classification

We identified eQTLs using eight distinct tissue types encompassing different developmental stages from germination to plant maturity (GRoot, Gshoot, Kern, L3Base, L3Tip, LMAD, LMAN, and seedling) (Bukowski et al., 2018; Kremling et al., 2018; Mazaheri et al., 2019). SNPs were filtered by removing non-biallelic markers, and those with a minor allele frequency (<0.05). Each of the tissue-specific expression datasets were filtered independently by retaining genes with ≥ 6 reads in ≥ 20% of samples and ≥ 0.1 TPM in ≥ 20% of samples. After filtering, we tested between 15.5M - 16.7M SNPs against the expression of 15.3K - 26.4K genes across the eight tissue types. Briefly, to test SNP-gene associations, a series of eight candidate linear models were fitted beginning with a naive t-test then progressively controlling for different levels of kinship and population structure in a mixed linear model. For each model tested, the association was deemed significant if the observed P-value surpassed the 10K permutation threshold computed for each gene. Non-significant eQTLs were discarded when the association was supported by fewer than two of the candidate linear models and when the association involved non-syntenic genes (Schnable, 2019). These associations were classified as cis-eQTLt, trans/cis-eQTL, cis-eQTL, trans-eQTL, and unassigned eQTL according with the distance between each eQTL and its corresponding target gene as well as its co-location with annotated maize genes (genome B73-V4, Figure S3.2a).

### Protein-DNA interactions data analysis

Raw reads from plant ChIP- (Bolduc et al., 2012; Morohashi et al., 2012; Eveland et al., 2014; Pautler et al., 2015; Li et al., 2018; Zhan et al., 2018; Dong et al., 2019), ChIP-seq from protoplast (pChIP-seq) (Tu et al., 2020), and DAP-seq (Ricci et al., 2019; Dong et al., 2020) were collected from publicly available datasets. Quality control of reads and peak identification was performed as reported previously (Gomez-Cano et al., 2022). Briefly, read quality control was performed using FastQC (http://www.bioinformatics.babraham.ac.uk/projects/fastqc/, V0.11.5). Adapters and low quality reads were trimmed with Trimmomatic (Bolger et al., 2014) using the following parameters: ILLUMINACLIP:Adapter.fastq:2:40:15 SLIDINGWINDOW:4:20 MINLEN:30. Cleaned reads were mapped to the B73 maize reference genome (AGPv4) (Jiao et al., 2017) with Bowtie2 v2.3.4.1 (Langmead and Salzberg, 2012) and only using nuclear chromosomes. Multi-mapping reads were filtered with Samtools v1.9 (Li et al., 2009) (q 30). Peaks were called using GEM v3.4 (Guo et al., 2012). In DAP-seq assays, sequences retrieved using the HALO protein alone were used as a control. Peaks from plant ChIP-seq experiments were called including duplicates and using the corresponding mutants or tag-protein as control. Finally, ChIP from protoplasts was called including replicates. All of them used the following parameters: --d Read_Distribution_default.txt --k_min 6 --k_max 15 --k_seqs 2000 --outNP --sl. Only TFs with >500 predicted peaks were used for further analysis.

#### Peak quality control.

We evaluated the overlapping of TF peaks with accessible chromatin regions (ACRs) and scale the number of CPM per peaks and assay. Thus, peaks with a Z-score larger than −0.5 and mapping to ACR were kept for further analysis. Specifically, to obtain CPMs per peak, we used the summit of each peak and extended them 50 bps around the summit. Then, we convert extended summit files into SAF files and count mapped reads using Rsubread v1.32.2 (Liao et al., 2019), followed by CPM normalization. Publicly available ACRs were collected from (Marand et al., 2021).

### Functional annotation

All functional annotations were performed after discarding non syntenic genes. PWYs were collected from CornCYC (Andorf et al., 2016), and GO terms were obtained from GAMER (Wimalanathan et al., 2018). Syntenic genes were defined based on Schnable et al. (2019). Enrichment analysis for PWYs and GO terms was conducted in R using the GeneOverlap (v1.30.0) and topGO (v2.46.0) packages, respectively. GO term semantic similarity was calculated using the GOSemSim (v2.20.0) package in R, employing the “Wang” method. For *common function* analysis, all GO comparisons were performed on the original set of enriched GO terms, and after the comparisons (GO semantic similarity), all GO terms were mapped to their closest parent GO terms using the R package Rrvgo (v1.6) (Sayols, 2023). Similarly, all GO terms significantly enriched from *common target* and *network-based* analyses were mapped to its closed parent before any description to reduce redundancy.

### Network integration.

#### Common interactions.

We compared TF-target associations across all layers (i.e., GAN, GRN, eGRN, and CEN networks) and considered interactions as common when they were present in at least two different layers for the same corresponding TF. Subsequently, we assessed the enrichment of PWYs and GO terms using the common TF interactions. Any significant GO terms were then mapped to their closest parent terms.

#### Common function.

Common functions were identified by testing the enrichment of target genes with PWYs and GO terms for each TF in each layer. TFs that had at least one PWY/GO term enriched in at least two different layers were retained to assess common predictions across layers (Figure S3.5a). The similarity in PWY predictions among layers was performed by comparing all PWYs between layers for each TF using a Fisher exact test. We used the enrichment test because a single gene could be annotated in multiple PWYs. The similarity in GO term predictions were performed by measuring the semantic similarity between the corresponding terms. Significant GO terms are then mapped to their closest parent terms.

#### Network-based.

All four layers were combined and then scaled the interaction frequencies from 0.5 to 1, as follow 0.5 + (0.5/4)*N, being N the number of times that same interaction was observed. Embeddings of the scaled network were identified with PecanPy (Liu and Krishnan, 2021) using the following parameters: --weighted --dimensions 50 --walk-length 80 --num-walks 10 --directed. Gene similarity was assessed by computing the mutual rank (MR) of the mutual information (MI) using the following formula: MR_MI_ = sqrt(MI_rank * tMI_rank), where MI_rank represents the rank of the MI matrix and tMI_rank represents the transpose matrix of MI_rank. The MI was calculated using the R package Parmigene (Sales and Romualdi, 2011). To select highly similar genes by TF based on its MR_MI_ we used a decay function as follows: D = *e*^−(MRMI −1)/50^. D values ≤ 0.05 were taken as highly similar (Wisecaver et al., 2017). After identifying genes highly similar per TF, we proceed to test the enrichment of PWY and GO terms. Significant GO terms are then mapped to their closest parent terms.

### Evaluation of functional predictions based in loss of function mutant and random network validation.

The PWY and GO term predictions were contrasted with the enriched PWY and GO terms identified in loss of function mutants (aka, knockout) using the differential express genes (DEGs) and their corresponding log2FC values by TF. We used data from previous studies for KN1 (Bolduc et al., 2012), RA1 (Eveland et al., 2014), FEA4 (Pautler et al., 2015), O2 (Zhan et al., 2018), bZIP22 (Li et al., 2018), and TB1 (Dong et al., 2019) reanalyzed in (Zhou et al., 2020). Additional data for MYBR32_m1, WRKY82_m1, HSF13m1m2, HSF18m1, HSF20m1, HSF29m1, HSF29m2, WRKY2m2, WRKY8m1, and WRKY8m2 were collected from (Ellison et al., 2023). The enrichment of PWY and GO terms were performed with DEG selected based on adjusted P-value as reported by DESeq2 (Padj ≤ 0.05) (Love et al., 2014) and following enrichment analyses described above (Methods Functional annotation). The similarities between PWYs and GO terms predicted and those observed in the corresponding knockout were estimated using PWY overlapping and GO semantic similarity, as previously described (Methods Functional annotation). Gene set enrichment analysis was conducted using the R package FGSEA (v1.20) (Korotkevich et al., 2021) with the parameters: minSize = 5, maxSize = 1000, and eps = 0. The gene sets tested were defined based on the predicted PWYs and GO terms for each TF, considering the available knockout data. The fraction of recovered predictions was calculated by determining the number of significant (P-value ≤ 0.05) PWYs and GO terms out of the total tested.

The comparison of each method’s prediction against the random networks was conducted by randomizing each of the four initial neworks (GRN, eGRN, CEN, and GAN) 3,000 times, generating 3,000 random versions of each network type. Subsequently, we annotated and integrated each set of random networks following the procedures described in Methods sections Network integration and Functional annotation. All random networks were generated using the “rewire” function from the R package Igraph (v1.2.4.1), with the following parameters: avoided loops and with niter = NodesInNetwork * 10000).

### Prioritization of transcriptional regulators-process associations.

All prioritization analyses were conducted using *network-based* results. GO terms with less than 800 genes were retained, and after mapping excessively specific GO terms (≤ 50 genes) to their corresponding GO terms parent. Mapping to parent terms was performed following the procedures described in about (Methods Functional annotation). Then, we proceeded to calculate the enrichment score associated with each TF-GO association as follow:

$$E_{ij}={Log}_{2}[(c/t)/(p/u)]$$

Where $E_{ij}$ is the enrichment score of the TFi with the GOj,c is the intersection of target genes of TFi and annotated genes on GOj,t is the total number of target genes of TFi,p is the total number of genes annotated on GOj, and u is the total number of genes in maize, which in this case refers to the total number of syntenic genes with sorghum (Schnable, 2019). All Eij values were subsequently normalized by each TFi and GOj as follows:

Zi=(Eij-Ui)/σi

and

Zj=(Eij-Uj)/σj

Here, Ui and Uj represent the average enrichment score value for all the GOj associated with TFi and all the TFi associated with GOj, respectively. Similarly, σi and σj represent the standard deviation of the enrichment score value for all the GOj associated with TFi and all the TFi associated with GOj, respectively. Finally, we calculated the reciprocal Z-score (rZ) as follow:

$$rZij=sqrt(max(0,Zi{)}^{\wedge 2}+max(0,Zj{)}^{\wedge 2})$$

### Similarities in sequence among TF paralogs

Sequences for all transcripts associated with the corresponding pair of paralgos were collected from MaizeGDB (https://maizegdb.org/) using genome v4 (Jiao et al., 2017). TF similarities were calculated by averaging the Hamming distance between all transcripts associated with the respective TFs. The Hamming distance was computed using the R package DECIPHER (v2.22) (Wright, 2016) and the “DistanceMatrix” function with the following parameters: includeTerminalGaps = TRUE, penalizeGapLetterMatches = TRUE, and correction = “none”.

### Mapping of TF-GO terms into specific conditions by gene set enrichment analysis (GSEA).

The GSEA test was performed as previously reported (Gomez-Cano et al., 2022). Briefly, GSEA was performed using R package FGSEA v1.18.0 (Korotkevich et al., 2021) and with Pearson Correlation Coefficients (PCCs) as scoring metric (Korotkevich et al., 2021). PCC was calculated as the weighted PCC (wPCC) between the TFs and the corresponding genes annotated with the corresponding GO term. The expression dataset and conditions were defined previously (*RNA-seq, and co-expression data*) and Zhou et al., (2022). Expression values (CPM) were log_2_ transformed, and wPCCs were calculated using the R package wCorr (Version 1.9.1) (Emad and Bailey, 2017) with an optimal threshold of 0.4. Finally, the genes by GO term were collected from GAMER (Wimalanathan et al., 2018).

## Supplementary Material

Supplement 1 Figure S1. Summary of total TFs and their interactions used in the co-expression network (CEN) layer. a. Histogram showing the frequency of TFs with at least a target gene per CEN. The dotted gray line indicates average TFs along all 46 CEN. b. Boxplot indicating total target genes per TFs across the different CENs. CEN are named following Zhou et al., (2022) nomenclature. Orange labels highlight TFs with the largest number of target genes in several CENs. c. Histogram with the frequency of total target genes per TF after combined results from all 46 CENs. Descriptions of CEN in (b) can be found in Table S3.
Figure S2. Defining a gene association network (GAN) based on *trans*-eQTL. a. model indicating total eQTLs identified and the classification schema used to define *trans*-eQTL, *trans*/*c*is-eQTL, *cis*-eQTLt, and *cis*-eQTL. Within them, trans-eQTLs were used to define the GAN. In the context of trans-eQTLs, a source gene (in blue) was defined as a gene whose promoter (2kb upstream from TSS) or gene body overlapped with an eQTL. Genes whose expression is explained by the SNP variation were defined as gene targets (gene in yellow). b and c. I classify each source and target gene into five functional categories to count the number of associations by category (unclassified genes defined as other). Left panel, Boxplot indicating the number of targets (b) and source (c) genes by each gene category. Right panel, Stacked bar plots indicate the fraction of each gene category over the total genes in GAN. d. Bar plot indicating total interactions by gene category pair.
Figure S3. Establishing the maize gene regulatory network (GRN) layer based on protein-DNA interaction data. a. Density plot with distribution of peaks by PDI data type. b and c. Stacked bar plot with fraction of peaks mapped to accessible chromatin region (ACR) (b) and with low peak coverage (c) CPM scaled and filtered; Z ≤ −0.5. d. Locally weighted scatterplot smoothing (LOESS) line plot of Zscores by peak in 10 kb bins around 200 kb of the closest transcription start site (TSS). e. Classification schema (top) and corresponding proportion of total combined peaks (bottom, first stacked bar plot) and peaks by method (bottom, second stacked bar plot) utilized for determining target genes.
Figure S4. Overview of strategy to annotate TFs based on common targets. a. Schema of pipeline used to annotate TFs based on common target genes amount layer (GAN, GRN, eGRN, and CEN). b and c. Venn diagram indicating the number of common TFs with at least one and ten target genes. d. Venn diagram indicating total common interactions (TF-target gene) among layers.
Figure S5. Overview of strategy to annotate TFs based on common functions. a. Schema of pipeline used to annotate TFs based on common function amount layer (GAN, GRN, eGRN, and CEN). b. Bar plot indicating total TFs annotated by layer and by type of function. c and d. Venn diagram indicating the number of TFs with at least a PWY (c) and GO term (d) commonly enriched among the corresponding layers.
Figure S6. Network-based strategy to annotate TFs. a. Schema of pipeline used on the integration of layers to identify TF with similar topological properties, defined here as network-based TF annotation. b. Histogram plot indicating the distribution of genes associated per TF. c. Stacked bar plot with total TFs annotated by enrichment with PWYs and GO terms. d. Bar plot indicating the percentage of TFs annotated for the 82 TF families (and co-regulator) with at least a TF annotated (c).
Figure S7. Predicted PWY overlapped poorly with PWY observed in knockouts assays. a. Heatmap shows the count of overlapped PWYs between predicted and observed PWY in knockouts per method. The Violet box signifies significant overlap (P-value 0.05, Fisher test). An empty box (white) denotes no predicted PWYs for the corresponding TF. Square braking indicates the number of PWYs significantly enriched in the corresponding knockout (P-value <= 0.05, Fisher test). b. Stacked bar plot indicating the fraction of predicted PWY significantly enriched on DEGs per knockout assay and method.
Figure S8. GO semantic similarities observed between predicted GO terms and enriched GO terms in knockout are not occurring by chance. Density plot displaying the distribution of random GO term semantic similarity. The observed value on real GO term enrichment, along with its corresponding P-value concerning the random distribution, is highlighted by the horizontal purple line.
Figure S9. Target genes and expression distribution of TFs compared with knockout results. a. Histogram and density plot display the scaled (Z-score) number of target genes for each layer. TFs utilized in the knockout analysis are indicated by dotted red lines. b. Heatmap shows the presence or absence of target genes in each of the four layers for every TF analyzed in the knockout assays. c. Histogram and density plot of Tau index distribution for TF 2,910 TFs annotated with at least PWY/GO term. TFs utilized in the knockout analysis are indicated by dotted purple lines. d. Histogram and density plot illustrate the null distribution of Tau after randomly sampling 13 TFs a thousand times. TFs used in the knockout analysis are represented by dotted purple lines. P-values were calculated using the null distribution as a reference.
Figure S10. Evaluation of TF functional annotation by contrasting predictions random networks. a, b, c, and d. Boxplot showing the fraction of random network with significantly enrich GO terms (a), average number of GO terms (b), -log10FDR (c), and GSS (d) observed in 3000 random networks by each method. The GSS values were calculated by comparing each random network with the observed GO terms from the true TF-target interactions. Asterisks indicate P-value significance (*: p ≤ 0.05, **: 633 p ≤ 0.01, ***: p ≤ 0.001, ****: p ≤ 0.0001, two-sided t-test).
Figure S11. GO term significance and similarity distributions from random networks per TF. a and b. Density ridges plot showing the average -log_10_FDR (a) and GSS (b) distributions in 3,00 random networks for each TF. The GSS values were calculated by comparing each random network with the observed GO terms from the true TF-target interactions.
Figure S12. Scale count of GO terms in random networks. Density plot displaying the distribution of GO terms in random networks predicted by the *network-based* method for each corresponding TF. The observed number of significantly enriched GO terms for the corresponding TF is indicated by a dotted orange line. The p-value was calculated using the random distribution as the null distribution.
Figure S13. Enrichment score for TF and GO term in several biological processes. a, b, and c. Scatter plot with reciprocal Z score (rZ) of hormones- (a), metabolism- (b), and development-related (c) process.
Figure S14. Differences in GSEA enrichment per TF’s cluster. a and b Boxplot with the percentage of co-expression networks (a) and the number of GO terms significantly enriched (FDR <= 0.1) per TF (b). c. Scatter plot indicating the total number of GO terms annotated by TF vs the percentage of GO terms co-expressed per TF. The color indicates the density of TFs overlapped. d, e, and f. Boxplot indicating the average percentage of significantly co-expressed GO term (d), number of networks with at least a significant GO term by TF, and (f) the number of GOs terms annotated per TF grouped in function of their GSEA enrichment similarities (Figure 4a).
Figure S15. GSEA enrichment profiles uncover TF-GO term regulatory effects which are condition-specific. a, b, and c. Heatmap indicates the normalized enrichment score (NES) between bHLH43, HB33, and WRKY25 and their corresponding GO term by co-expression network. NESs were calculated only indicated in networks where the corresponding TFs are expressed.
Supplementary data 1: Expanded descriptions of the TF functional annotation based on Common interactions and Common function.
Supplementary data 2: Evaluation of functional prediction by comparing with random networks.

Supplement 2 Table S1. List of inbred lines and their corresponding tissue used in the eQTL analysis.

Supplement 3 Table S2. Summary of ChIP- and DAP-seq publicly available reanalyzed in this work.

Supplement 4 Table S3. Description of datasets used as part of the co-expression networks.

Supplement 5 Table S4. List of expression datasets used in the identification of eQTLs.

Supplement 6 Table S5. Categories of genes used to define types of *trans*-eQTLs.

Supplement 7 Table S6. List of *trans*-eQTLs association identified for HSF20.

Supplement 8 Table S8. List of annotated functions (GO term and PWY) per TF and the method used on the corresponding annotation.

Supplement 9 Table S9. List of DEG per experiments used to evaluate TF annotation predicted in this work.

Supplement 10 Table S7. Total PDI (peaks) identify and used in the construction of the GRN.
