# Supplementary material for: Prioritizing Metabolic Gene Regulators through Multi-Omic Network Integration in Maize
Source: bioRxiv. 2024 Feb 27:2024.02.26.582075. Preprint. [Version 1] doi: 10.1101/2024.02.26.582075 (PMC10925184; doi:10.1101/2024.02.26.582075)
Supplement: Supplementary file 1 [file media-1.pdf]

Prioritizing Maize Metabolic Gene Regulators through Multi-Omic Network Integration in Maize

Fabio Gomez-Cano, Jonas Rodriguez, Peng Zhou, Yi-Hsuan Chu, Erika Magnusson, Lina Gomez-Cano, Arjun Krishnan, Nathan M Springer, Natalia de Leon, Erich Grotewold

SUPPLEMENTAL INFORMATION

Supplemental information includes:

Supplementary data 1

Supplementary data 2

Supplementary figures 1 to 15

Supplementary data

Supplementary data 1: Expanded descriptions of the TF functional annotation based on *Common interactions* and *Common function*.

Common interactions. To identify common interactions, we compared all layers with each other (Figure S4a), and obtained ~4.6M TF-target interactions adding up interactions from all four networks (Figure S4d, adding up all interactions). As expected, GRN and eGRN were the layers with the largest number of overlapping interactions (~112.5K), followed by GRN and CEN (~102.7K) (Figure S4d). After identifying interactions common to multiple layers (present in at least two networks), we kept 206.2k out of the 4.6M interactions, comprising 934 TFs and ~20.6K target genes (Figure S4d). Using target genes as a proxy to annotate TFs functions, we tested the enrichment of common target genes with PWYs and GO terms for each TF (See *Methods*). We included co-regulators in the analysis and treated them without distinction from TFs. We found 11,362 significant associations between TF and biological processes (TF-process), including 2,812 associations between TFs and PWY (TF-PWY) and 8,550 between TFs and GOs (TF-GO) [False Discovery Rate (FDR) ≤ 0.1, Fisher's Exact Test] (Figure 2a). On average, we identified ~8 PWYs and ~80 GO terms by TF (Figure 2b, boxplot yellow). Combining the PWY and GO term results, we annotated 347 TFs, out of which 235 TFs showed enrichment only in the PWYs analysis (Table S8). The remaining 112 TFs showed enrichment with both PWYs and GO terms (Figure 2c).

Common function. To identify common functions, we initially tested the enrichment of target genes with PWYs and GO terms for each TF in each layer. We retained TFs that had at least one PWY/GO term enriched in the last two different layers. This allowed us to explore common predictions between layers for the corresponding TFs (Figure S5a). We observed a variable number of TFs enriched with PWYs (ranging from 120 to 2,019 TFs) and GO terms (ranging from 72 to 1,777 TFs) across the different layers. Between the layers, eGRN had the fewest annotations, while CEN had the largest number of annotations (Figure S5b). Thus, after selecting TFs with at least one PWY and/or GO enrichment, we restricted our analysis to 966 TFs and 245 TFs, respectively. When considering PWY annotations, the layer pair of CEN & GAN had the highest number of TFs annotated (888 TFs), while the layer pair of GRN & GAN had the lowest

(59 TFs). Similarly, for GO term annotations, CEN & GAN had the highest number (130 TFs), while GRN & GAN had the lowest number (59 TFs) (Figure S5c, d). Regarding *common predictions*, we identified overlapping PWYs by evaluating gene overlap between all PWYs enriched per TF between layers (P-value ≤ 0.05, Fisher's Exact) (Figure S5a). A similar approach was used to identify common functions at the GO term level. However, due to the hierarchical and redundant nature of the GO terms, we employed semantic similarity rather than gene overlap to determine common GO terms per TF between layers (FDR ≤ 0.1) (See *Methods*). These two annotation analyses together yielded 7,081 TF-process annotations (727 TF-PWY and 6,354 TF-GO) (Table S8) (Figure 2a). On average, this corresponds to 3.5 different PWYs and 57.7 different GO term associations per TF (Figure 2b). In terms of TFs, these associations encompass annotations for 204 TFs through PWY enrichment and 110 TFs through GO term enrichment (Figure 2c).

Supplementary data 2: Evaluation of functional prediction by comparing with random networks

Despite recovering GO terms similar to - and enrichment with - GO terms from knockouts (Figure 2d,e), we were interested in determining which method generated the fewest false positives. Consequently, we evaluated the identification of GO terms from ~3,000 random networks to determine which method recovered the lowest fraction of false positives (See *Methods*). We counted the number and significance of the GO terms enriched in random networks as a measure of the precision, and the similarity of observed GOs (true TF-target interactions) with the GOs from random networks as measure of the accuracy of the corresponding methods. Additionally, to compare predictions across methods for the same TF, we restricted our analysis to the 32 TFs with GO predictions in all three methods (Figure 2c, green and blue intersection). We posited that methods with fewer GO terms, less significant FDR values, and GO terms from random networks less similar to observed GO terms are indicative of better predictions. Consistent with our results, the *network-based* method identified significantly enriched GO terms in only ~12% of random networks tested, which was substantially lower, compared to the ~28% and ~72% obtained with the *common function* and the *common target* methods, respectively (Figure S10a). Concordantly, *network-based* predicted significantly fewer GO terms (Figure S10b), with less significant P-values (Figure S10c), and GO terms

less similar (lowest GSS values) to the predicted from true interactions per TF than those observed with *common function* and *common target* methods (Figure S10d), highlighting that the *network-based* method produced the highest precision and accuracy. Notably, *the common function* method predicted fewer GO terms per TFs than the *common target* method. However, the predictions had P-values with higher statistical significance, and GSS values that were on par with those observed with the *common target* method (Figure S10). For any individual TF, we observed a high degree of overlap in the GSS distributions of the *common functional* and the *common target* methods, suggesting the two methods were equally noisy.

Overall, the *network-based* method detected a lower number of GO terms per TF (Figure 2b) and had GO terms enriched in significantly fewer random networks (Figure S10a). This suggests limitations in the method's ability to identify GO term associations, as it inherently identifies fewer GO terms per TF. To examine this possibility, we asked whether the number of GO terms observed with true interactions could be attributed to chance. We found that 30 out of the 32 tested TFs exhibited a significantly higher total number of GO terms compared to those expected by chance (P-value <= 0.05) (Figure S12). Thus, despite the *network-based* approach yielding fewer GO terms per TF, the identified GO terms contain curated and biologically meaningful information that is unlikely to occur due to chance alone. In conclusion, within the given data/layers context, the *network-based* method is a superior approach. Consequently, we exclusively relied on network-based predictions for subsequent analyses.

Supplementary Figures

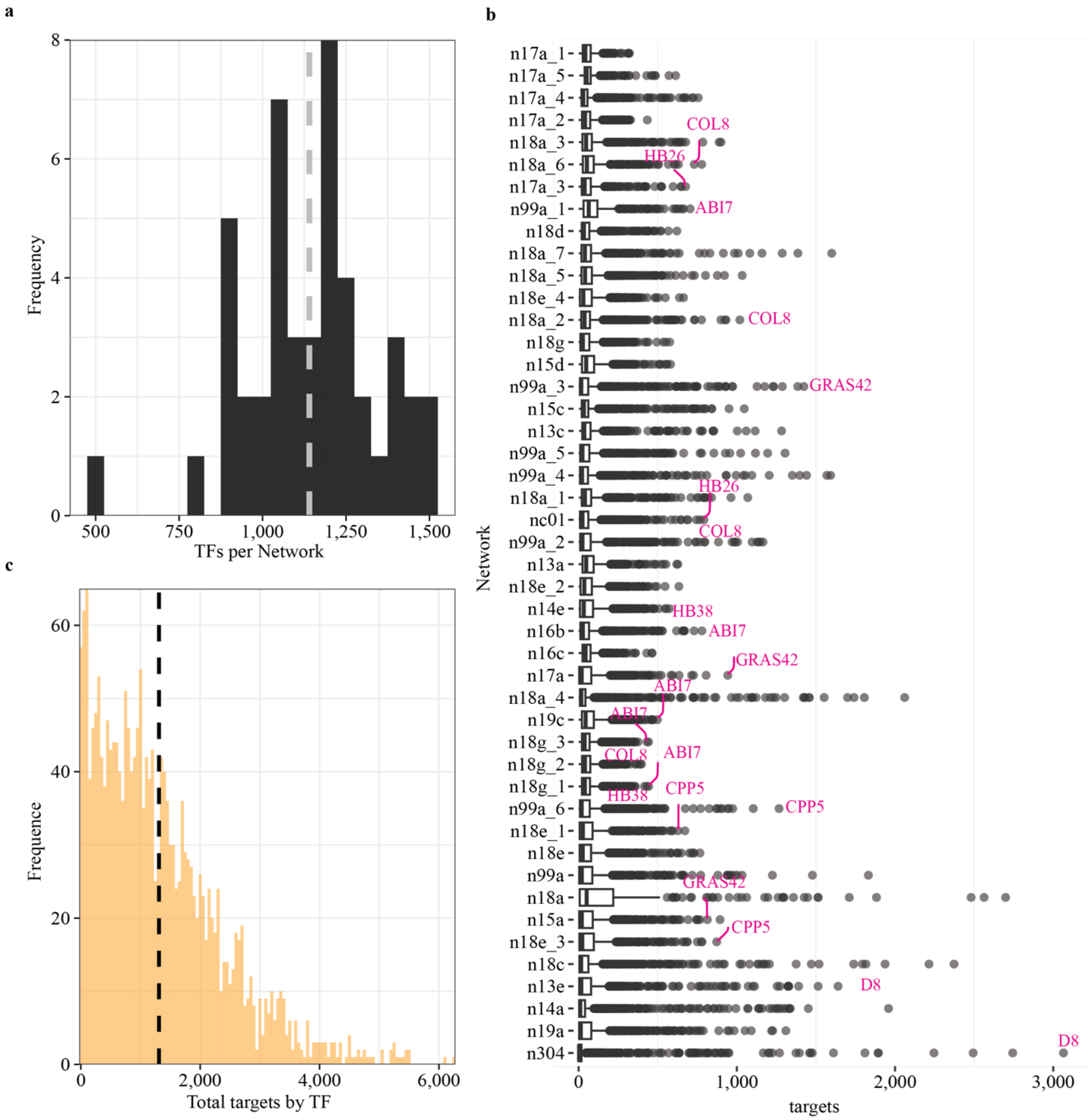

Figure S1.

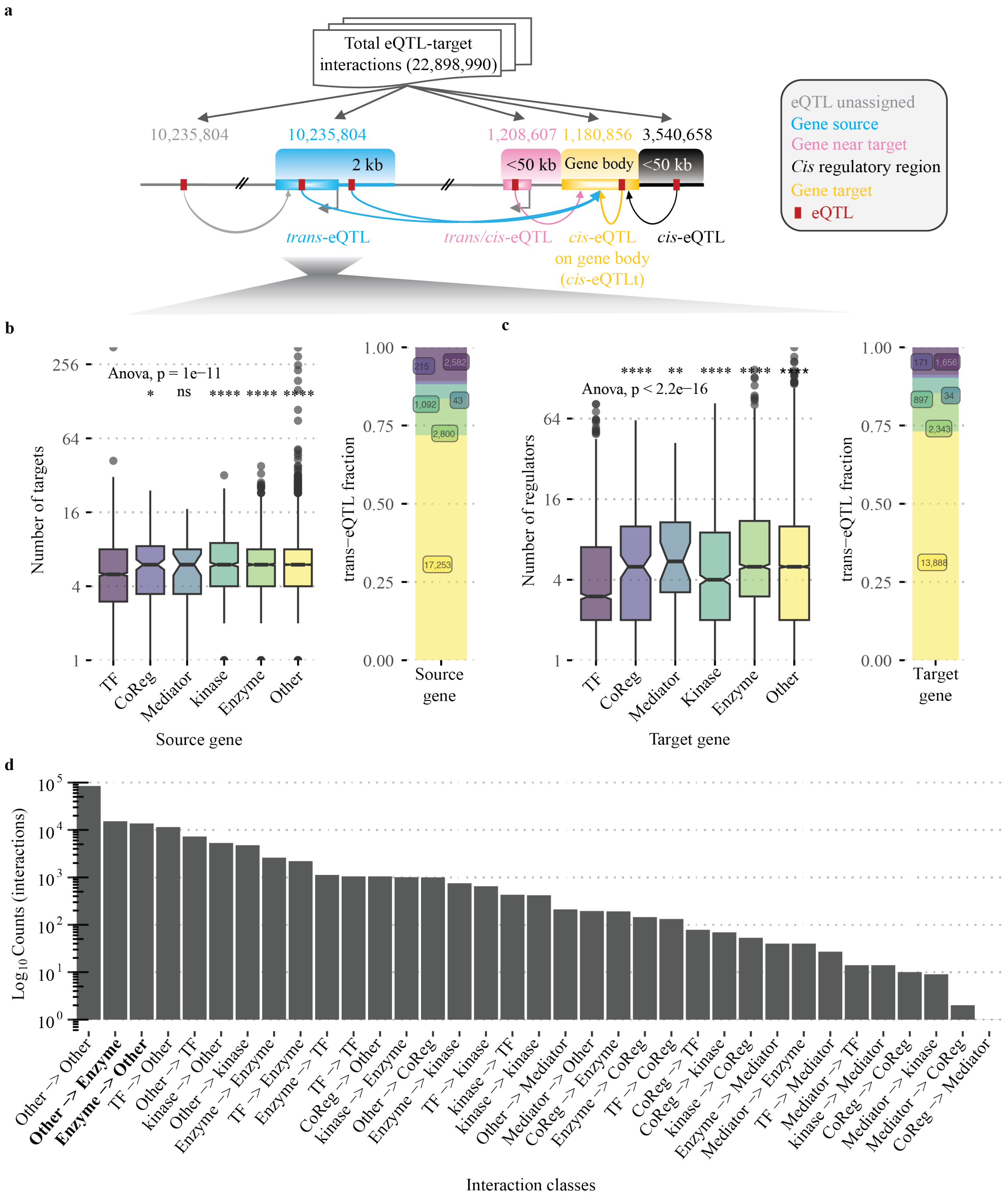

a
Total eQTL-target interactions (22,898,990)
10,235,804
10,235,804
1,208,607
1,180,856
3,540,658
2 kb
<50 kb
Gene body
<50 kb
trans-eQTL
trans/cis-eQTL
cis-eQTL on gene body (cis-eQTLt)
cis-eQTL
eQTL unassigned
Gene source
Gene near target
Cis regulatory region
Gene target
eQTL
b
Anova, p = 1e−11
Number of targets
Source gene
TF
CoReg
Mediator
kinase
Enzyme
Other
trans−eQTL fraction
Source gene
c
Anova, p < 2.2e−16
Number of regulators
Target gene
TF
CoReg
Mediator
Kinase
Enzyme
Other
trans−eQTL fraction
Target gene
d
Log10 Counts (interactions)
Interaction classes
Other -> Other
Other -> Enzyme
Enzyme -> Other
TF -> Other
Other -> TF
kinase -> Other
Other -> kinase
Enzyme -> Enzyme
TF -> Enzyme
Enzyme -> TF
TF -> TF
CoReg -> Other
kinase -> Enzyme
Other -> CoReg
Enzyme -> kinase
TF -> kinase
kinase -> TF
kinase -> kinase
Other -> Mediator
Mediator -> Other
CoReg -> Enzyme
Enzyme -> CoReg
TF -> CoReg
CoReg -> TF
CoReg -> kinase
kinase -> CoReg
Enzyme -> Mediator
Mediator -> Enzyme
TF -> Mediator
Mediator -> TF
kinase -> Mediator
CoReg -> CoReg
Mediator -> kinase
Mediator -> CoReg
CoReg -> Mediator

Figure S2.

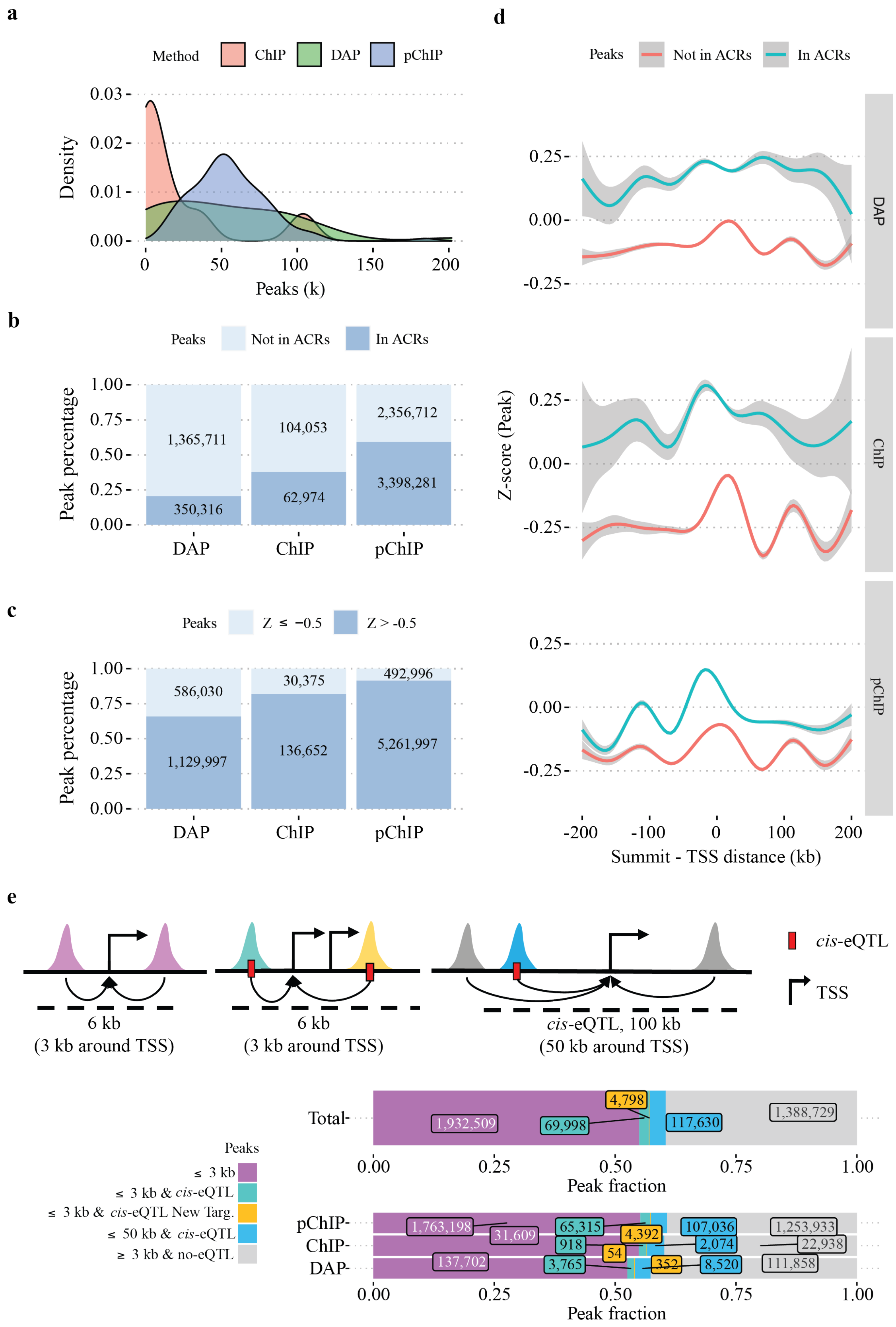

a
Method
ChIP
DAP
pChIP
Density
Peaks (k)
b
Peaks
Not in ACRs
In ACRs
Peak percentage
1,365,711
350,316
104,053
62,974
2,356,712
3,398,281
DAP
ChIP
pChIP
c
Peaks
Z ≤ −0.5
Z > -0.5
586,030
1,129,997
30,375
136,652
492,996
5,261,997
d
Peaks
Not in ACRs
In ACRs
Z-score (Peak)
Summit - TSS distance (kb)
e
6 kb
(3 kb around TSS)
6 kb
(3 kb around TSS)
cis-eQTL, 100 kb
(50 kb around TSS)
cis-eQTL
TSS
Peaks
≤ 3 kb
≤ 3 kb & cis-eQTL
≤ 3 kb & cis-eQTL New Targ.
≤ 50 kb & cis-eQTL
≥ 3 kb & no-eQTL
Total
1,932,509
69,998
4,798
117,630
1,388,729
Peak fraction
pChIP
ChIP
DAP
1,763,198
31,609
65,315
4,392
107,036
1,253,933
918
54
2,074
22,938
137,702
3,765
352
8,520
111,858

Figure S3.

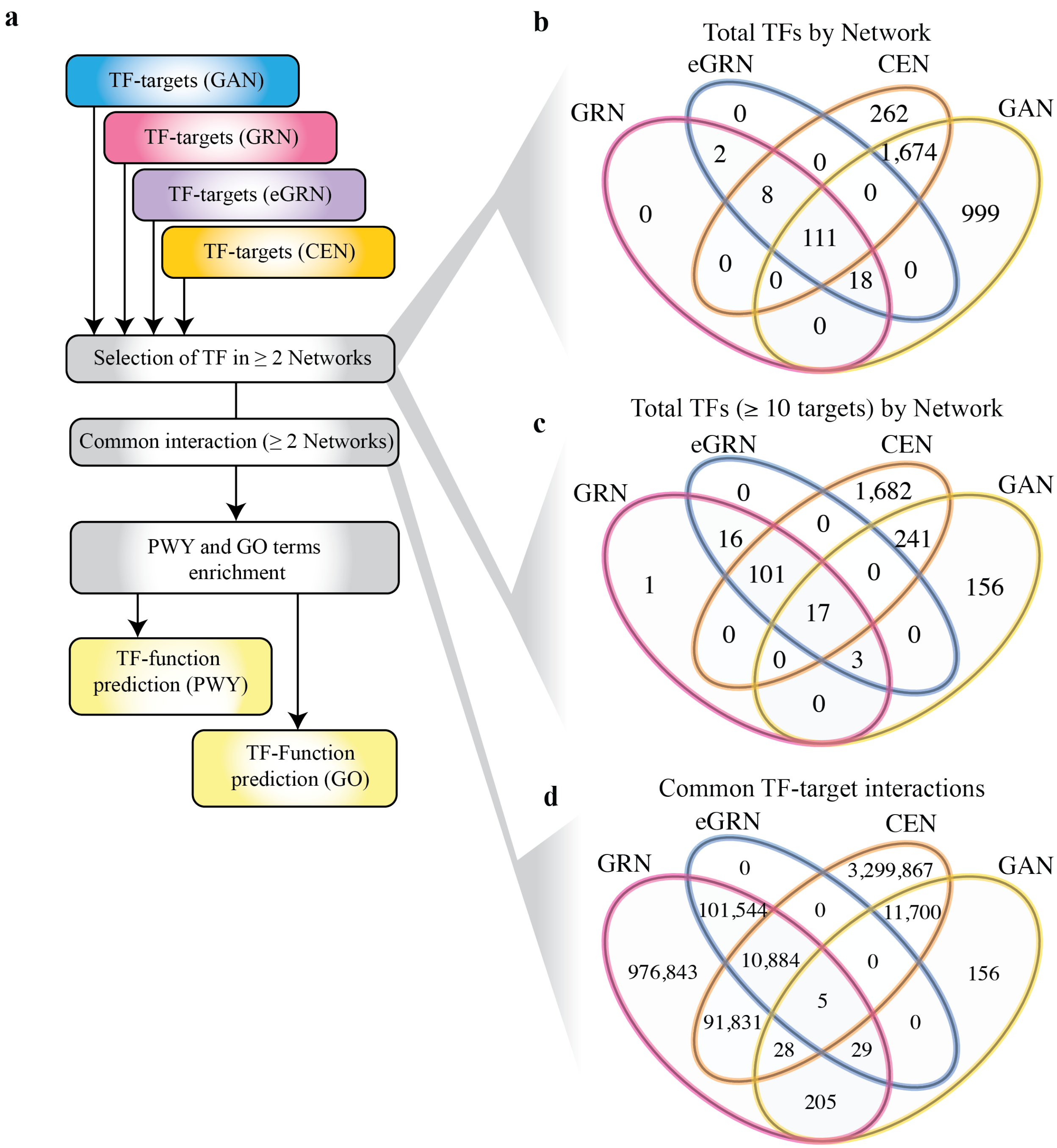
a
TF-targets (GAN)
TF-targets (GRN)
TF-targets (eGRN)
TF-targets (CEN)
Selection of TF in ≥ 2 Networks
Common interaction (≥ 2 Networks)
PWY and GO terms enrichment
TF-function prediction (PWY)
TF-Function prediction (GO)
b
Total TFs by Network
eGRN
CEN
GRN
GAN
0
262
2
0
1,674
8
0
0
999
111
0
0
18
0
0
c
Total TFs (≥ 10 targets) by Network
eGRN
CEN
GRN
GAN
0
1,682
16
0
241
101
0
1
156
17
0
0
0
3
0
d
Common TF-target interactions
eGRN
CEN
GRN
GAN
0
3,299,867
101,544
0
11,700
10,884
0
976,843
156
5
91,831
0
28
29
205

Figure S4.

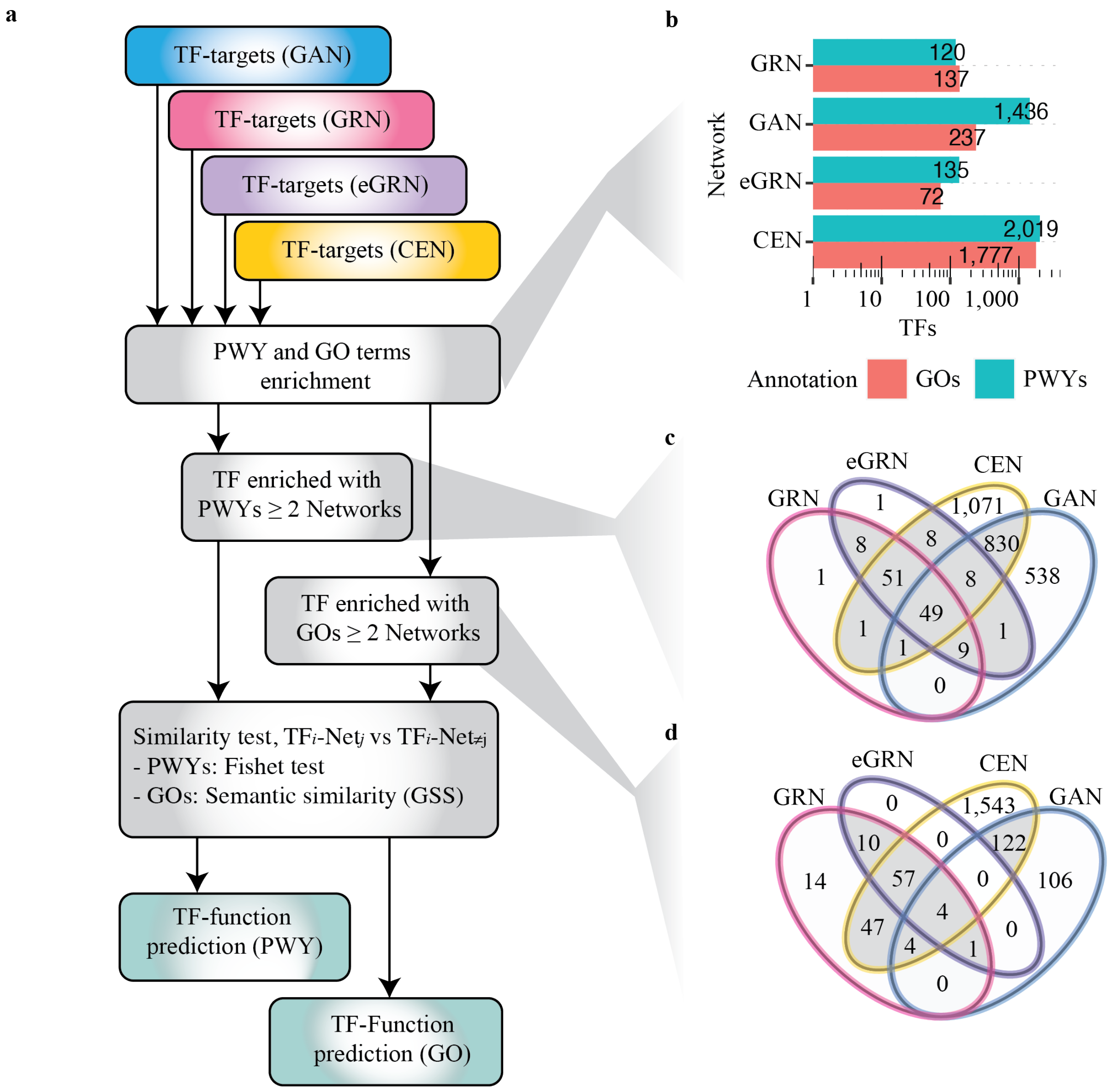
a
TF-targets (GAN)
TF-targets (GRN)
TF-targets (eGRN)
TF-targets (CEN)
PWY and GO terms enrichment
TF enriched with PWYs ≥ 2 Networks
TF enriched with GOs ≥ 2 Networks
Similarity test, TFi-Netj vs TFi-Net≠j
- PWYs: Fishet test
- GOs: Semantic similarity (GSS)
TF-function prediction (PWY)
TF-Function prediction (GO)
b
GRN
GAN
eGRN
CEN
Network
120
137
1,436
237
135
72
2,019
1,777
1
10
100
1,000
TFs
Annotation
GOs
PWYs
c
GRN
eGRN
CEN
GAN
1
1,071
8
8
830
1
51
8
538
49
1
1
1
9
0
d
GRN
eGRN
CEN
GAN
0
1,543
10
0
122
14
57
0
106
4
47
0
4
1
0

Figure S5.

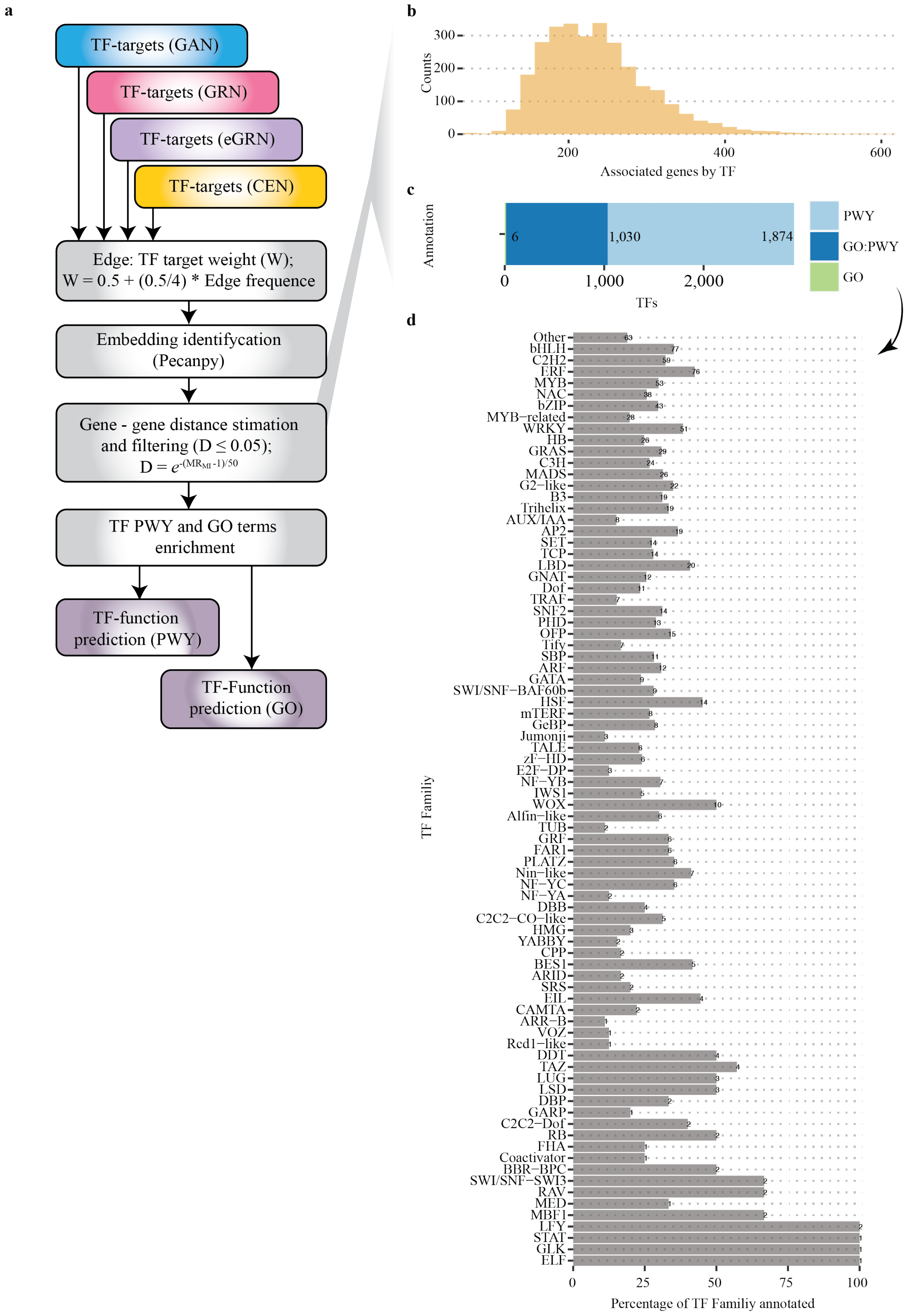
a
TF-targets (GAN)
TF-targets (GRN)
TF-targets (eGRN)
TF-targets (CEN)
Edge: TF target weight (W);
W = 0.5 + (0.5/4) * Edge frequence
Embedding identifycation
(Pecanpy)
Gene - gene distance stimation
and filtering (D ≤ 0.05);
D = e^-(MR_MI -1)/50
TF PWY and GO terms
enrichment
TF-function
prediction (PWY)
TF-Function
prediction (GO)
b
Counts
Associated genes by TF
c
Annotation
6
1,030
1,874
TFs
PWY
GO:PWY
GO
d
TF Familiy
Percentage of TF Familiy annotated
Other
bHLH
C2H2
ERF
MYB
NAC
bZIP
MYB−related
WRKY
HB
GRAS
C3H
MADS
G2−like
B3
Trihelix
AUX/IAA
AP2
SET
TCP
LBD
GNAT
Dof
TRAF
SNF2
PHD
OFP
Tify
SBP
ARF
GATA
SWI/SNF−BAF60b
HSF
mTERF
GeBP
Jumonji
TALE
zF−HD
E2F−DP
NF−YB
IWS1
WOX
Alfin−like
TUB
GRF
FAR1
PLATZ
Nin−like
NF−YC
NF−YA
DBB
C2C2−CO−like
HMG
YABBY
CPP
BES1
ARID
SRS
EIL
CAMTA
ARR−B
VOZ
Rcd1−like
DDT
TAZ
LUG
LSD
DBP
GARP
C2C2−Dof
RB
FHA
Coactivator
BBR−BPC
SWI/SNF−SWI3
RAV
MED
MBF1
LFY
STAT
GLK
ELF

Figure S6.

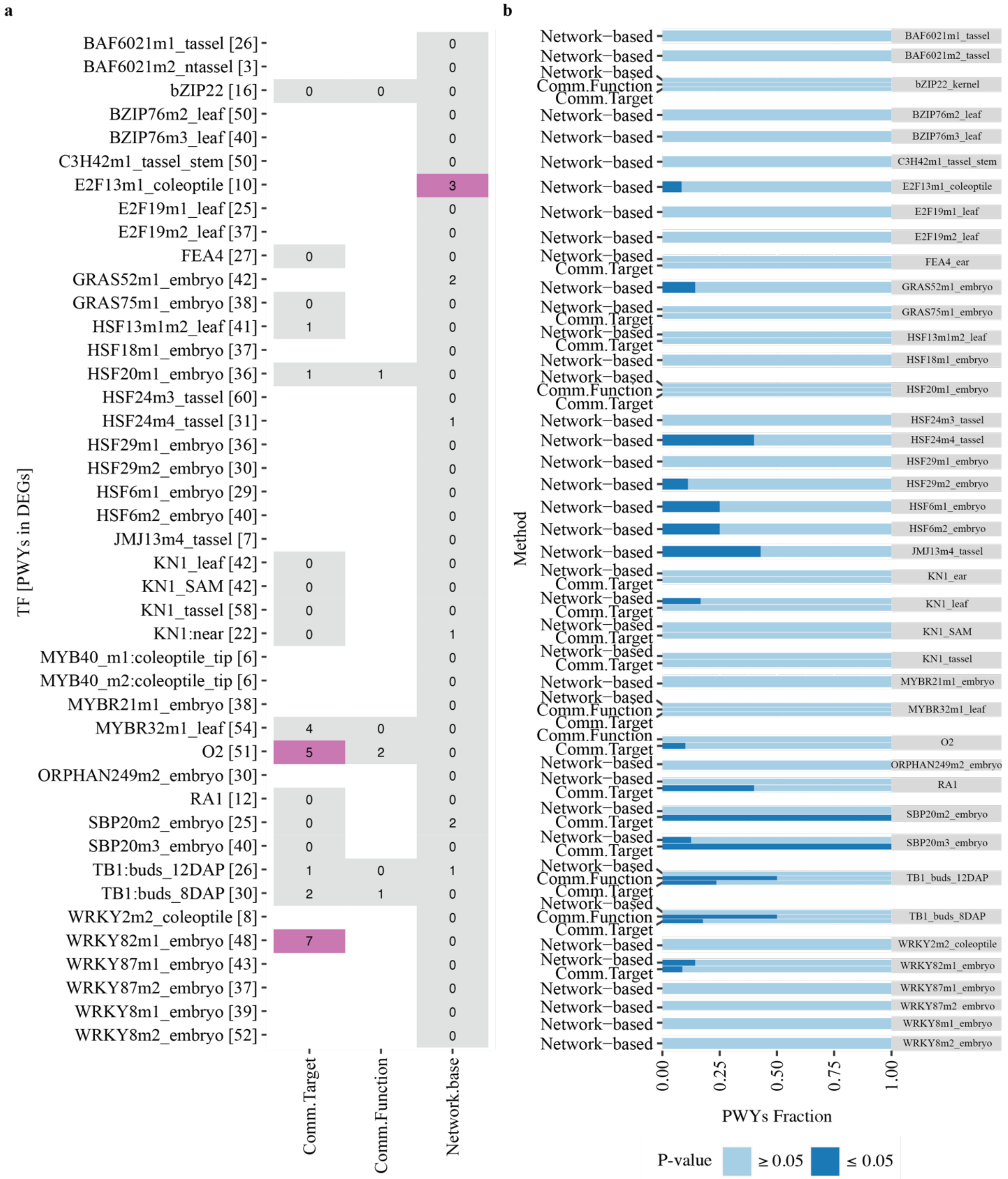

a
b
TF [PWYs in DEGs]
BAF6021m1_tassel [26]
BAF6021m2_ntassel [3]
bZIP22 [16]
BZIP76m2_leaf [50]
BZIP76m3_leaf [40]
C3H42m1_tassel_stem [50]
E2F13m1_coleoptile [10]
E2F19m1_leaf [25]
E2F19m2_leaf [37]
FEA4 [27]
GRAS52m1_embryo [42]
GRAS75m1_embryo [38]
HSF13m1m2_leaf [41]
HSF18m1_embryo [37]
HSF20m1_embryo [36]
HSF24m3_tassel [60]
HSF24m4_tassel [31]
HSF29m1_embryo [36]
HSF29m2_embryo [30]
HSF6m1_embryo [29]
HSF6m2_embryo [40]
JMJ13m4_tassel [7]
KN1_leaf [42]
KN1_SAM [42]
KN1_tassel [58]
KN1:near [22]
MYB40_m1:coleoptile_tip [6]
MYB40_m2:coleoptile_tip [6]
MYBR21m1_embryo [38]
MYBR32m1_leaf [54]
O2 [51]
ORPHAN249m2_embryo [30]
RA1 [12]
SBP20m2_embryo [25]
SBP20m3_embryo [40]
TB1:buds_12DAP [26]
TB1:buds_8DAP [30]
WRKY2m2_coleoptile [8]
WRKY82m1_embryo [48]
WRKY87m1_embryo [43]
WRKY87m2_embryo [37]
WRKY8m1_embryo [39]
WRKY8m2_embryo [52]
Comm.Target
Comm.Function
Network.base
Method
Network−based
Comm.Function
Comm.Target
PWYs Fraction
0.00
0.25
0.50
0.75
1.00
P-value
≥ 0.05
≤ 0.05

Figure S7.

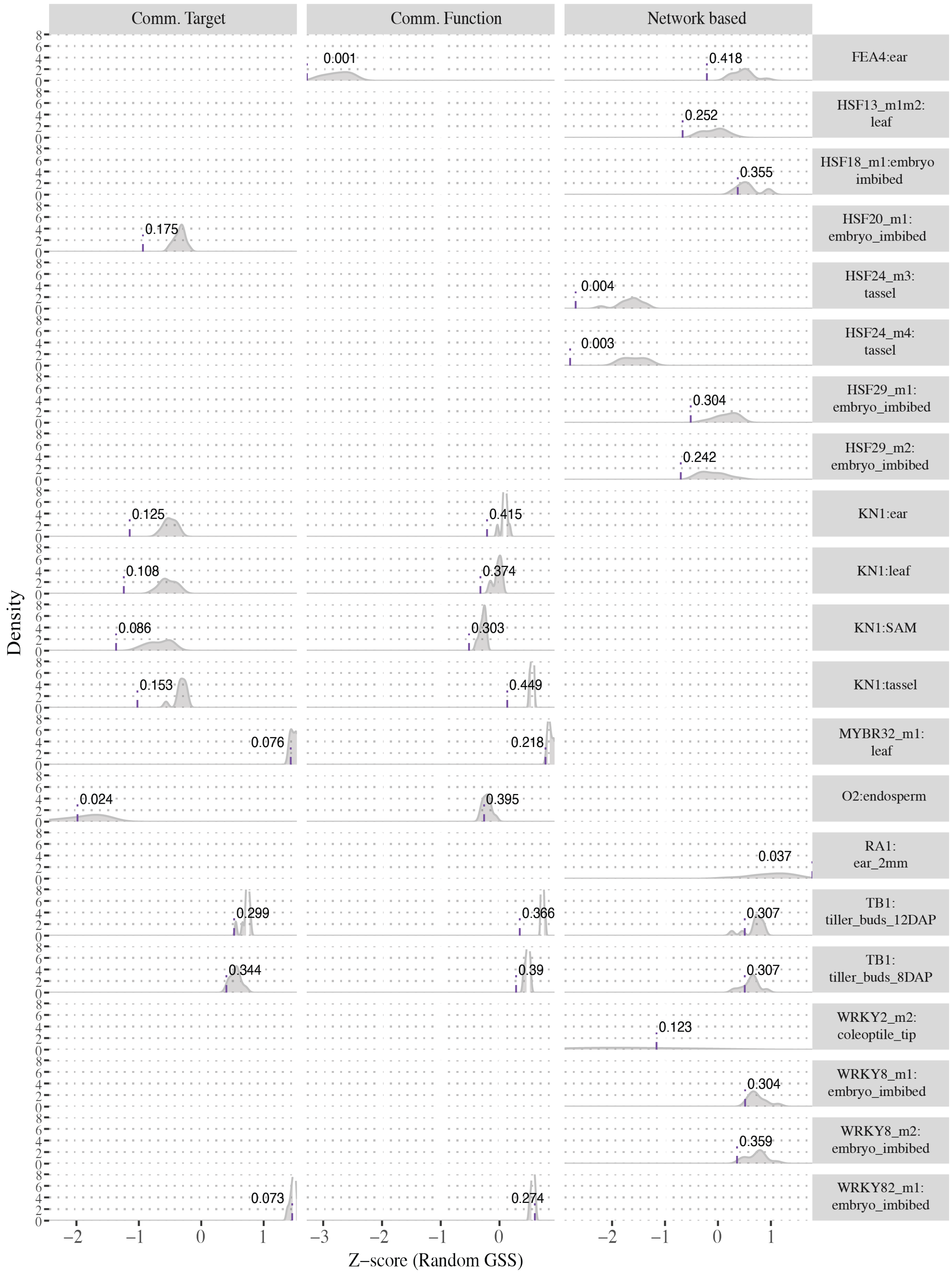
Comm. Target
Comm. Function
Network based
FEA4:ear
HSF13_m1m2: leaf
HSF18_m1:embryo imbibed
HSF20_m1: embryo_imbibed
HSF24_m3: tassel
HSF24_m4: tassel
HSF29_m1: embryo_imbibed
HSF29_m2: embryo_imbibed
KN1:ear
KN1:leaf
KN1:SAM
KN1:tassel
MYBR32_m1: leaf
O2:endosperm
RA1: ear_2mm
TB1: tiller_buds_12DAP
TB1: tiller_buds_8DAP
WRKY2_m2: coleoptile_tip
WRKY8_m1: embryo_imbibed
WRKY8_m2: embryo_imbibed
WRKY82_m1: embryo_imbibed
0.001
0.418
0.252
0.355
0.175
0.004
0.003
0.304
0.242
0.125
0.415
0.108
0.374
0.086
0.303
0.153
0.449
0.076
0.218
0.024
0.395
0.037
0.299
0.366
0.307
0.344
0.39
0.307
0.123
0.304
0.359
0.073
0.274
Density
Z−score (Random GSS)

Figure S8.

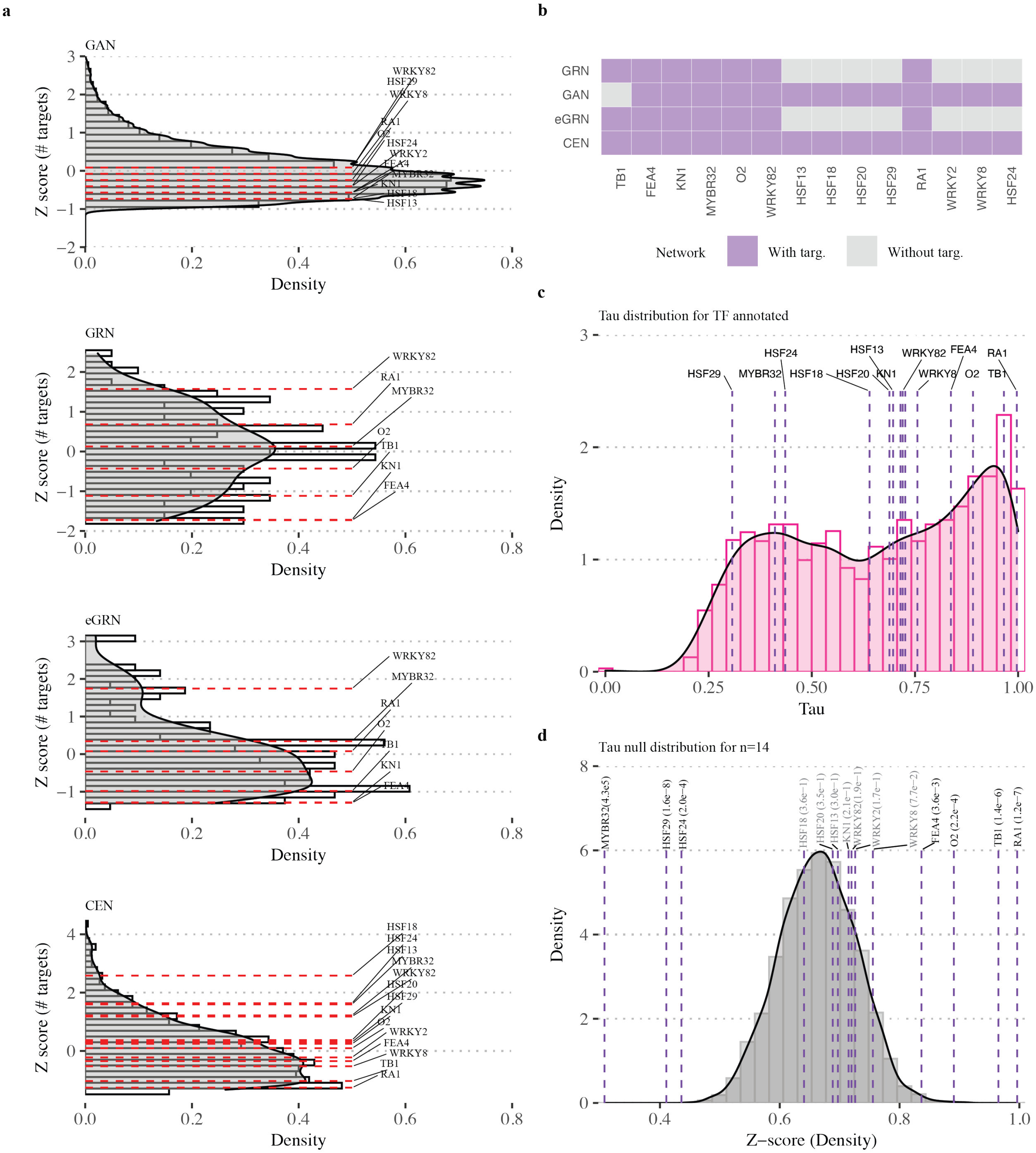
a
GAN
GRN
eGRN
CEN
Z score (# targets)
Density
b
GRN
GAN
eGRN
CEN
TB1 FEA4 KN1 MYBR32 O2 WRKY82 HSF13 HSF18 HSF20 HSF29 RA1 WRKY2 WRKY8 HSF24
Network
With targ.
Without targ.
c
Tau distribution for TF annotated
Tau
Density
d
Tau null distribution for n=14
Z−score (Density)
Density
MYBR32(4.3e5)
HSF29 (1.6e−8)
HSF24 (2.0e−4)
HSF18 (3.6e−1)
HSF20 (3.5e−1)
HSF13 (3.0e−1)
KN1 (2.1e−1)
WRKY82(1.9e−1)
WRKY2(1.7e−1)
WRKY8 (7.7e−2)
FEA4 (3.6e−3)
O2 (2.2e−4)
TB1 (1.4e−6)
RA1 (1.2e−7)

Figure S9.

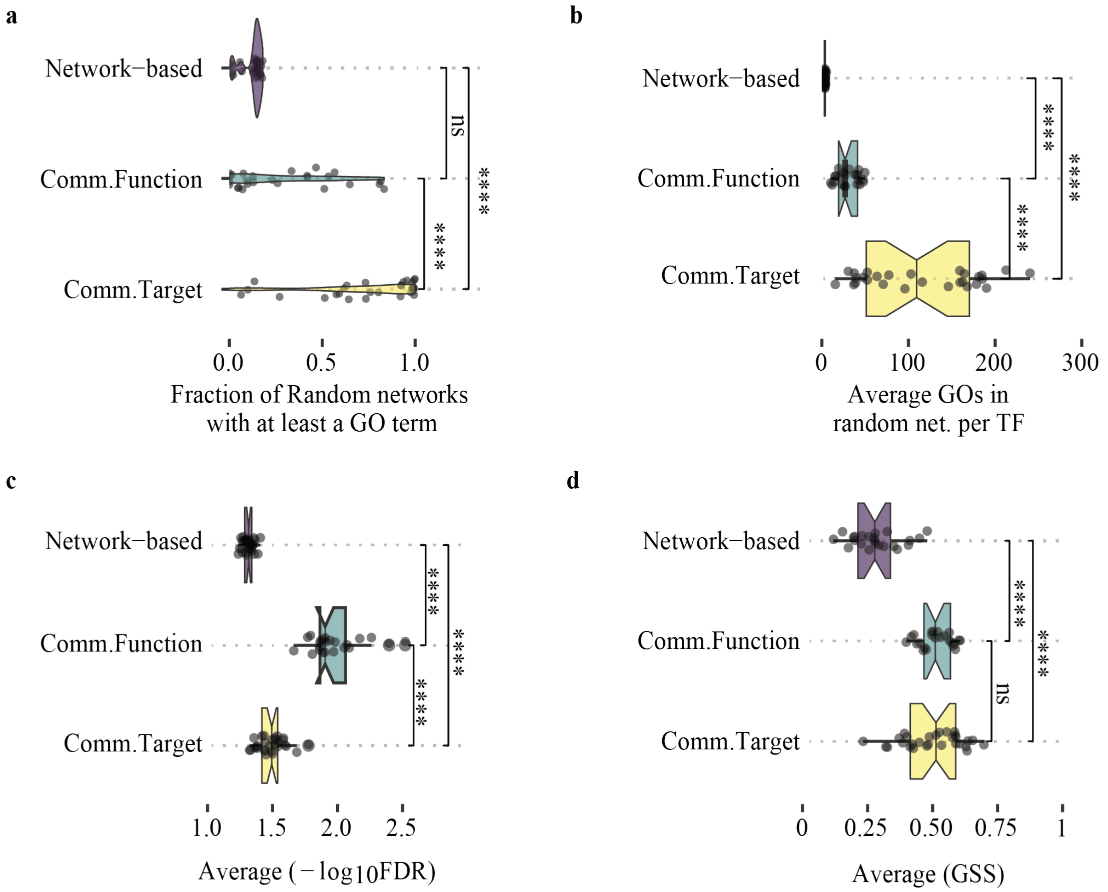
a
Network−based
Comm.Function
Comm.Target
ns
****
****
0.0
0.5
1.0
Fraction of Random networks with at least a GO term
b
Network−based
Comm.Function
Comm.Target
****
****
****
0
100
200
300
Average GOs in random net. per TF
c
Network−based
Comm.Function
Comm.Target
****
****
****
1.0
1.5
2.0
2.5
Average (−log10FDR)
d
Network−based
Comm.Function
Comm.Target
****
****
ns
0
0.25
0.50
0.75
1
Average (GSS)

Figure S10.

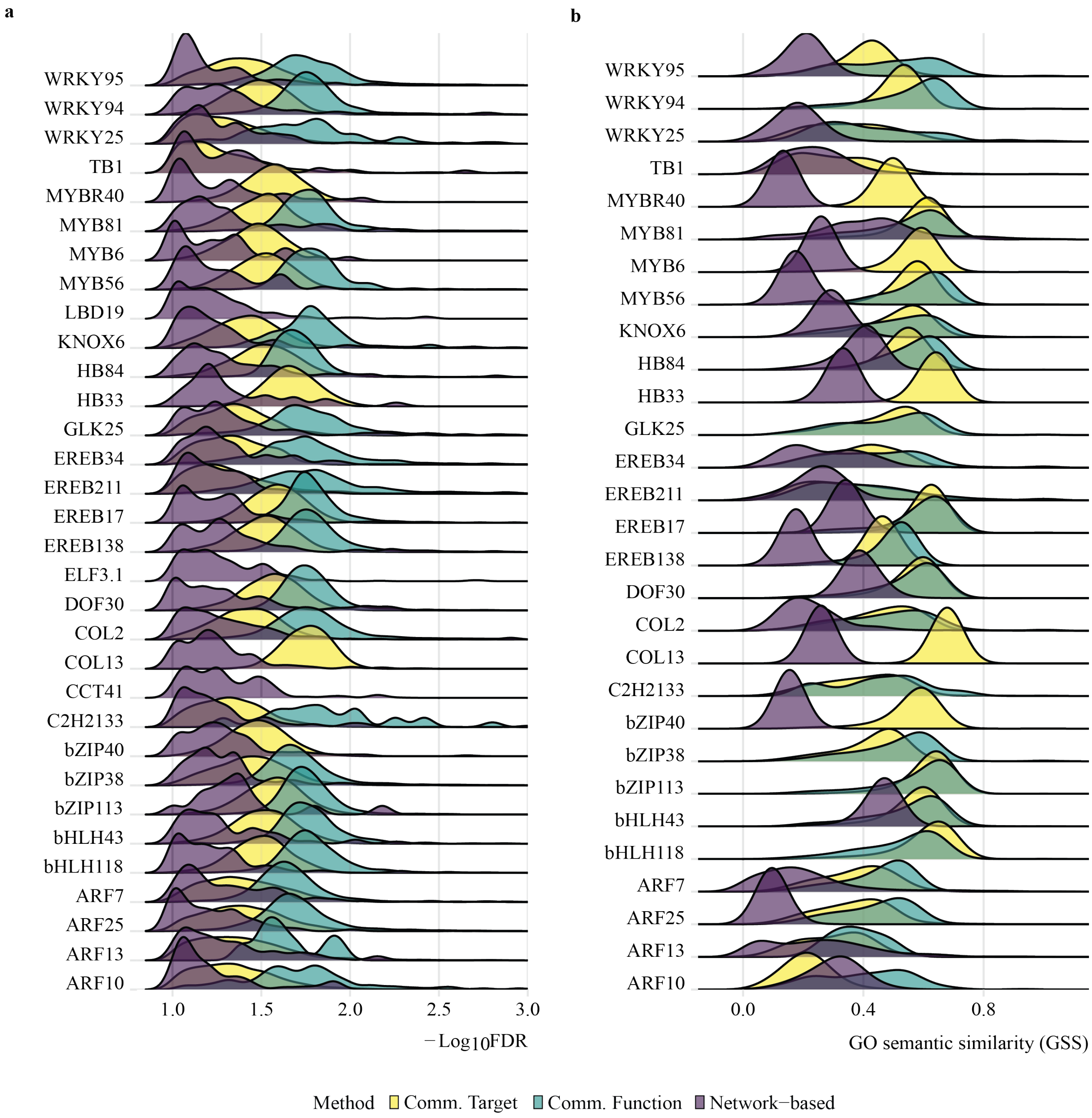

a
b
WRKY95
WRKY94
WRKY25
TB1
MYBR40
MYB81
MYB6
MYB56
LBD19
KNOX6
HB84
HB33
GLK25
EREB34
EREB211
EREB17
EREB138
ELF3.1
DOF30
COL2
COL13
CCT41
C2H2133
bZIP40
bZIP38
bZIP113
bHLH43
bHLH118
ARF7
ARF25
ARF13
ARF10
1.0
1.5
2.0
2.5
3.0
−Log10FDR
0.0
0.4
0.8
GO semantic similarity (GSS)
Method
Comm. Target
Comm. Function
Network−based

Figure S11.

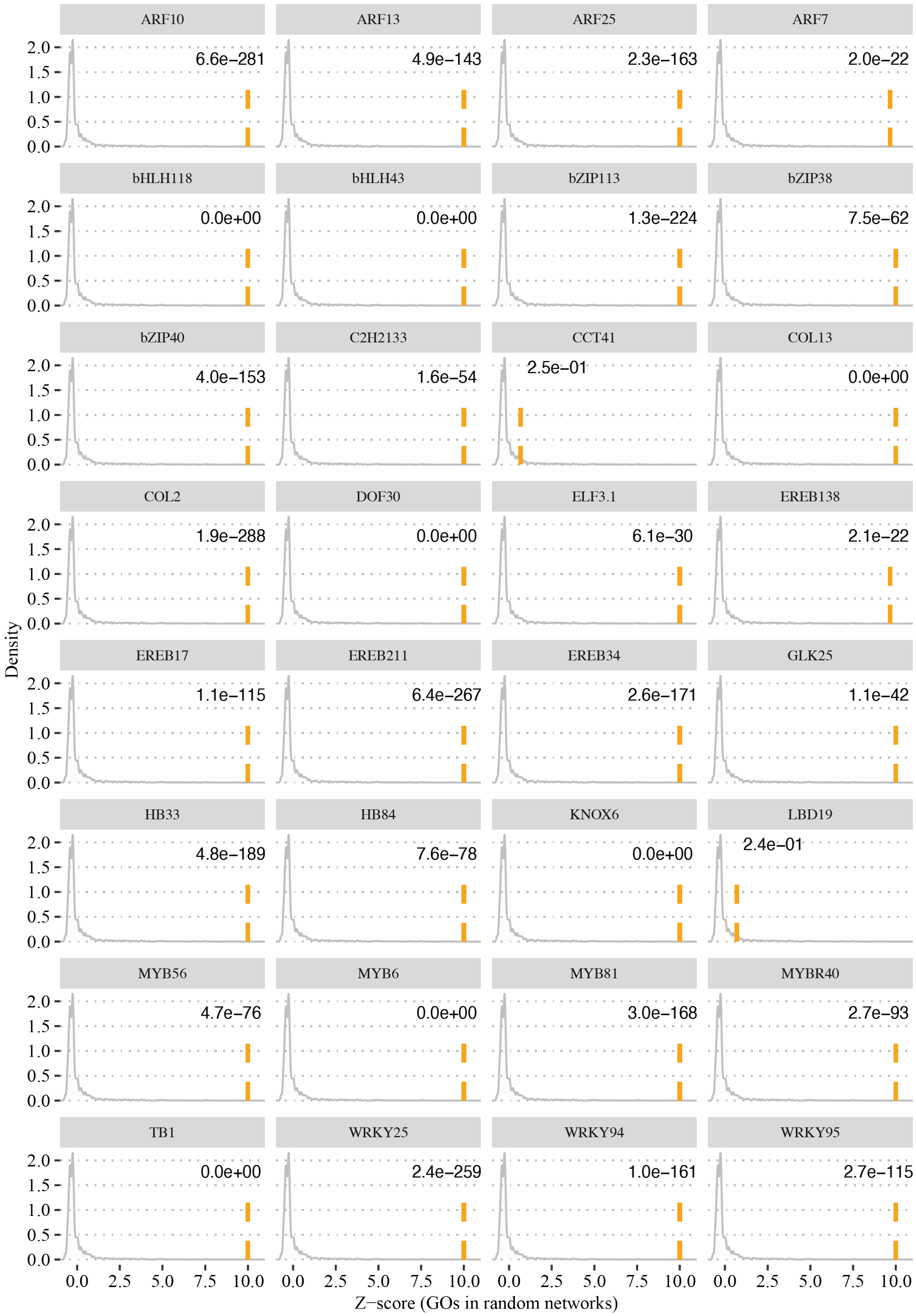
ARF10
6.6e−281
ARF13
4.9e−143
ARF25
2.3e−163
ARF7
2.0e−22
bHLH118
0.0e+00
bHLH43
0.0e+00
bZIP113
1.3e−224
bZIP38
7.5e−62
bZIP40
4.0e−153
C2H2133
1.6e−54
CCT41
2.5e−01
COL13
0.0e+00
COL2
1.9e−288
DOF30
0.0e+00
ELF3.1
6.1e−30
EREB138
2.1e−22
EREB17
1.1e−115
EREB211
6.4e−267
EREB34
2.6e−171
GLK25
1.1e−42
HB33
4.8e−189
HB84
7.6e−78
KNOX6
0.0e+00
LBD19
2.4e−01
MYB56
4.7e−76
MYB6
0.0e+00
MYB81
3.0e−168
MYBR40
2.7e−93
TB1
0.0e+00
WRKY25
2.4e−259
WRKY94
1.0e−161
WRKY95
2.7e−115
Density
Z−score (GOs in random networks)
0.0
0.5
1.0
1.5
2.0
2.5
5.0
7.5
10.0

Figure S12.

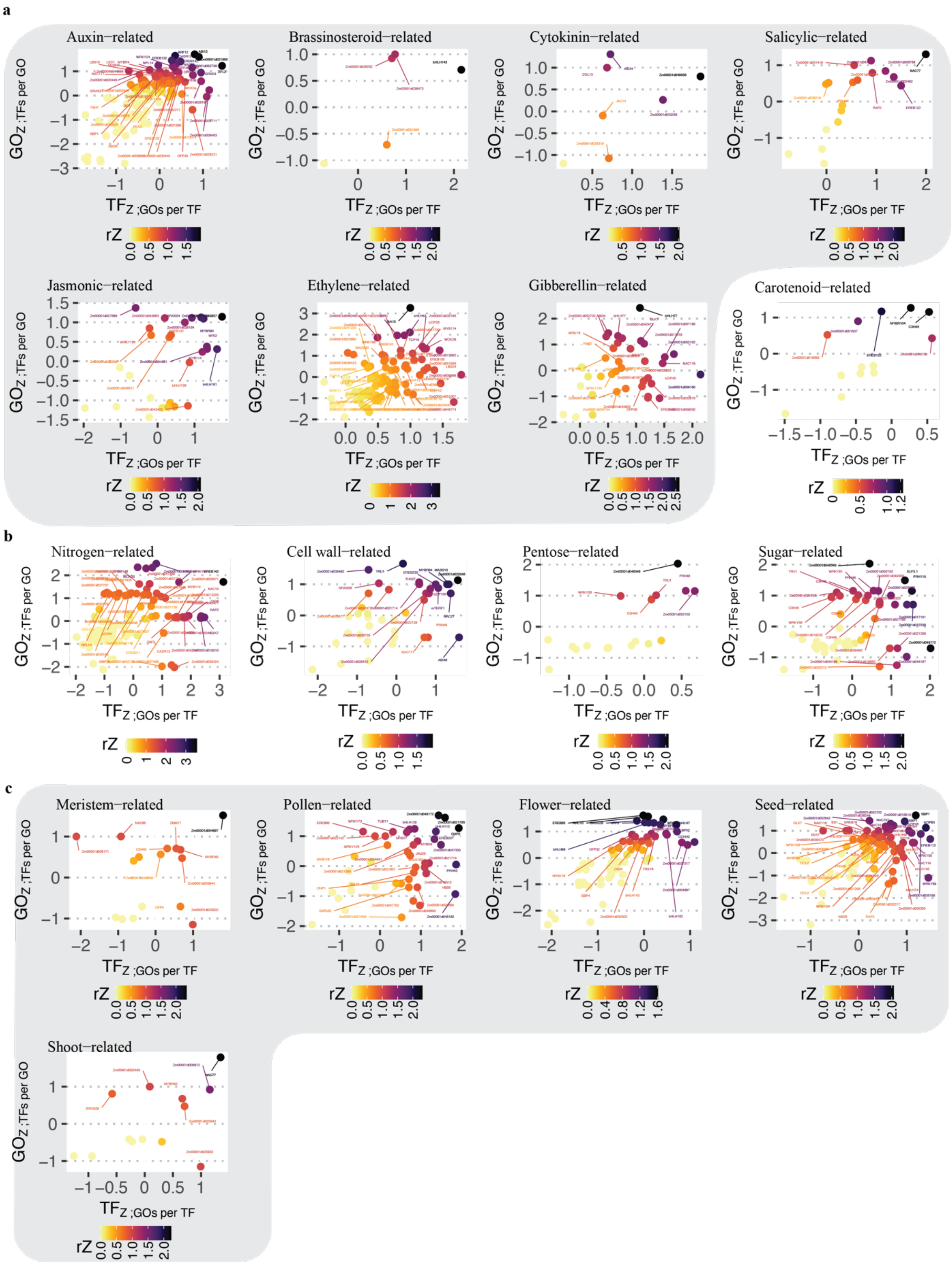
a
Auxin−related
Brassinosteroid−related
Cytokinin−related
Salicylic−related
Jasmonic−related
Ethylene−related
Gibberellin−related
Carotenoid−related
b
Nitrogen−related
Cell wall−related
Pentose−related
Sugar−related
c
Meristem−related
Pollen−related
Flower−related
Seed−related
Shoot−related
GOZ ;TFs per GO
TFZ ;GOs per TF
rZ

Figure S13.

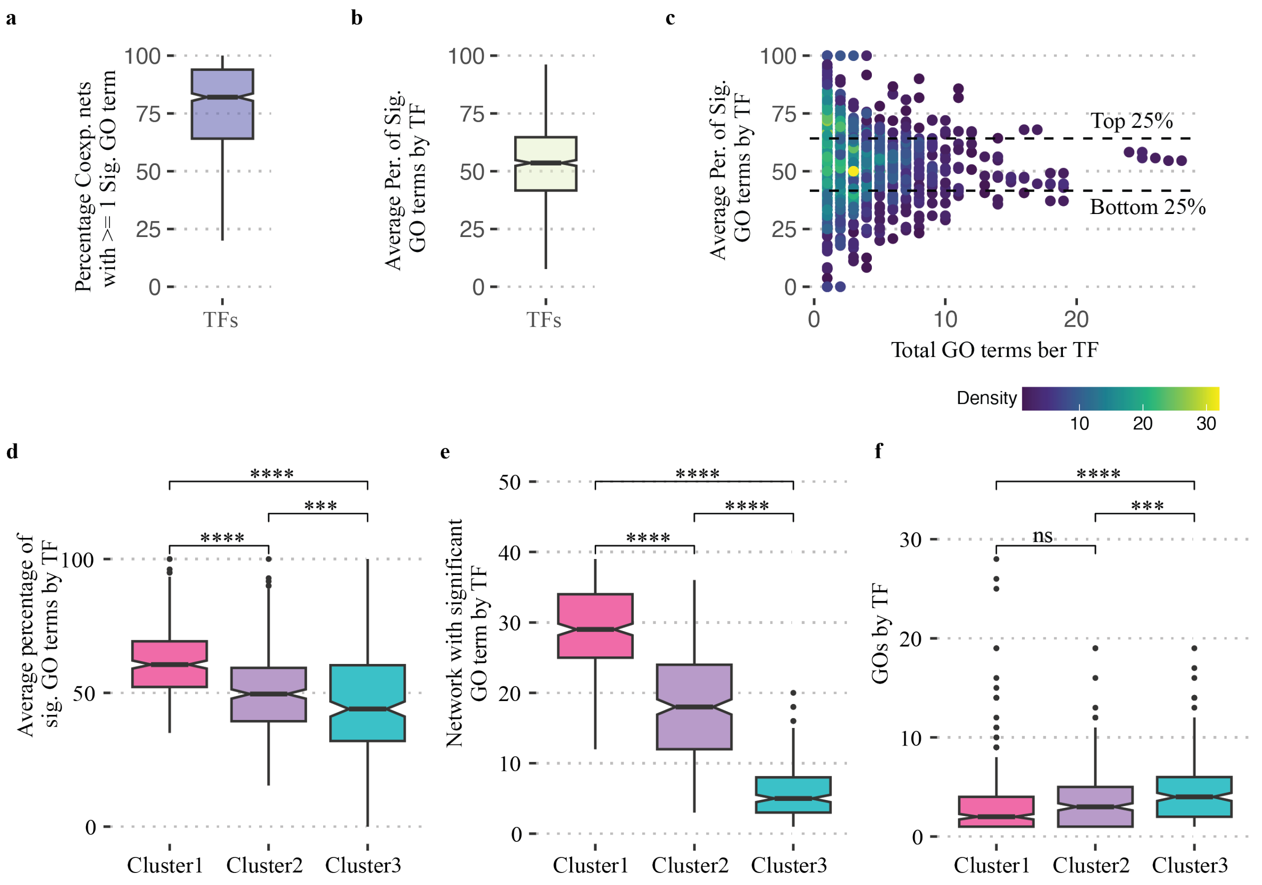
a
Percentage Coexp. nets with >= 1 Sig. GO term
TFs
b
Average Per. of Sig. GO terms by TF
TFs
c
Average Per. of Sig. GO terms by TF
Top 25%
Bottom 25%
Total GO terms ber TF
Density
d
Average percentage of sig. GO terms by TF
Cluster1
Cluster2
Cluster3
e
Network with significant GO term by TF
Cluster1
Cluster2
Cluster3
f
GOs by TF
ns
Cluster1
Cluster2
Cluster3

Figure S14.

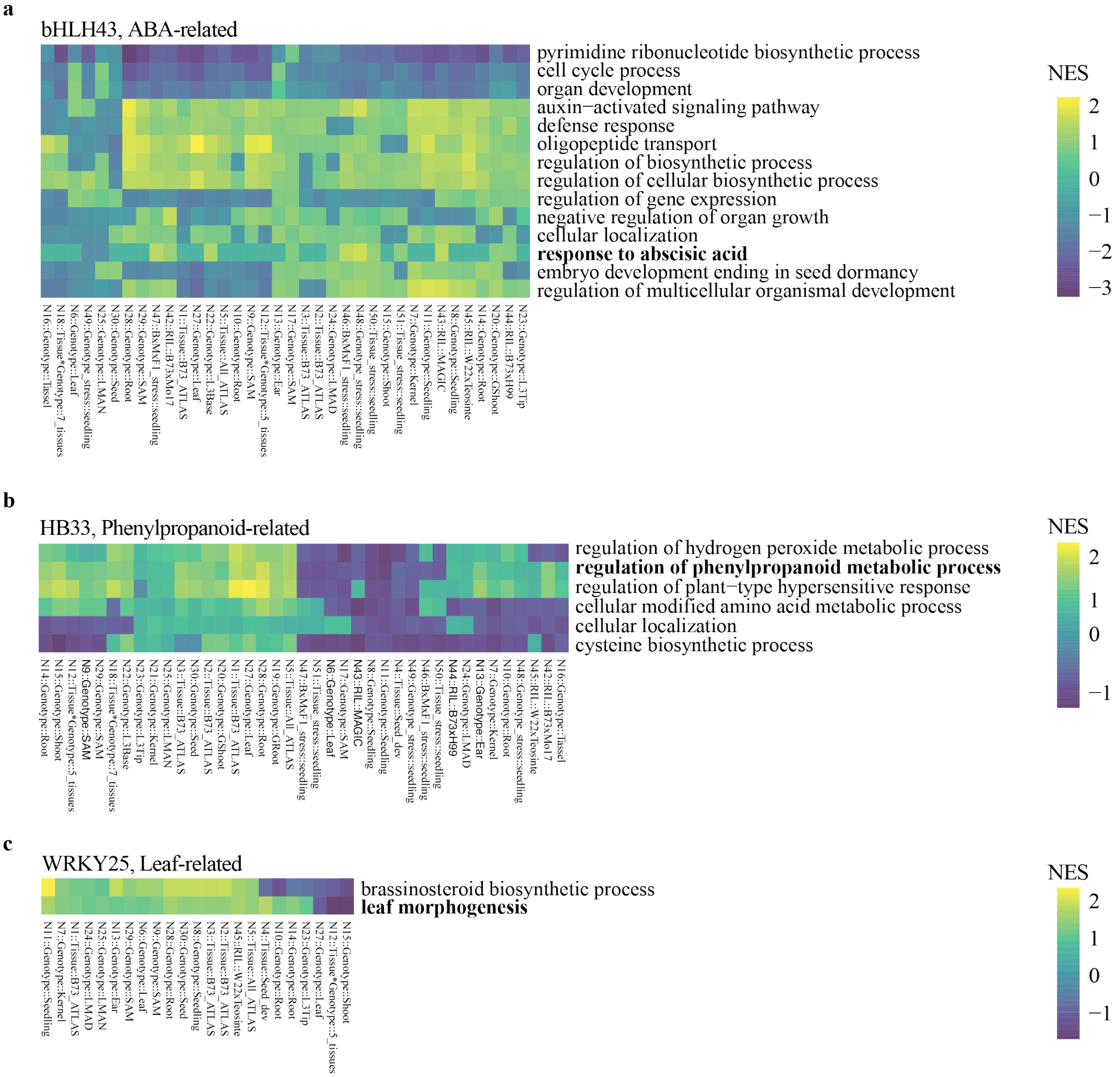
a
bHLH43, ABA-related
pyrimidine ribonucleotide biosynthetic process
cell cycle process
organ development
auxin−activated signaling pathway
defense response
oligopeptide transport
regulation of biosynthetic process
regulation of cellular biosynthetic process
regulation of gene expression
negative regulation of organ growth
cellular localization
response to abscisic acid
embryo development ending in seed dormancy
regulation of multicellular organismal development
N16::Genotype::Tassel
N18::Tissue*Genotype::7_tissues
N6::Genotype::Leaf
N49::Genotype_stress::seedling
N25::Genotype::LMAN
N30::Genotype::Seed
N28::Genotype::Root
N29::Genotype::SAM
N47::BxMxF1_stress::seedling
N42::RIL::B73xMo17
N1::Tissue::B73_ATLAS
N27::Genotype::Leaf
N22::Genotype::L3Base
N5::Tissue::All_ATLAS
N10::Genotype::Root
N9::Genotype::SAM
N12::Tissue*Genotype::5_tissues
N13::Genotype::Ear
N17::Genotype::SAM
N3::Tissue::B73_ATLAS
N2::Tissue::B73_ATLAS
N24::Genotype::LMAD
N46::BxMxF1_stress::seedling
N48::Genotype_stress::seedling
N50::Tissue_stress::seedling
N15::Genotype::Shoot
N51::Tissue_stress::seedling
N7::Genotype::Kernel
N11::Genotype::Seedling
N43::RIL::MAGIC
N45::RIL::W22xTeosinte
N8::Genotype::Seedling
N14::Genotype::Root
N20::Genotype::GShoot
N44::RIL::B73xH99
N23::Genotype::L3Tip
NES
2
1
0
−1
−2
−3
b
HB33, Phenylpropanoid-related
regulation of hydrogen peroxide metabolic process
regulation of phenylpropanoid metabolic process
regulation of plant−type hypersensitive response
cellular modified amino acid metabolic process
cellular localization
cysteine biosynthetic process
N14::Genotype::Root
N15::Genotype::Shoot
N12::Tissue*Genotype::5_tissues
N9::Genotype::SAM
N29::Genotype::SAM
N18::Tissue*Genotype::7_tissues
N22::Genotype::L3Base
N23::Genotype::L3Tip
N21::Genotype::Kernel
N25::Genotype::LMAN
N3::Tissue::B73_ATLAS
N30::Genotype::Seed
N20::Genotype::GShoot
N2::Tissue::B73_ATLAS
N1::Tissue::B73_ATLAS
N27::Genotype::Leaf
N28::Genotype::Root
N19::Genotype::GRoot
N5::Tissue::All_ATLAS
N47::BxMxF1_stress::seedling
N51::Tissue_stress::seedling
N6::Genotype::Leaf
N17::Genotype::SAM
N43::RIL::MAGIC
N8::Genotype::Seedling
N11::Genotype::Seedling
N4::Tissue::Seed_dev
N49::Genotype_stress::seedling
N46::BxMxF1_stress::seedling
N50::Tissue_stress::seedling
N44::RIL::B73xH99
N24::Genotype::LMAD
N13::Genotype::Ear
N7::Genotype::Kernel
N10::Genotype::Root
N48::Genotype_stress::seedling
N45::RIL::W22xTeosinte
N42::RIL::B73xMo17
N16::Genotype::Tassel
NES
2
1
0
−1
c
WRKY25, Leaf-related
brassinosteroid biosynthetic process
leaf morphogenesis
N11::Genotype::Seedling
N7::Genotype::Kernel
N1::Tissue::B73_ATLAS
N24::Genotype::LMAD
N25::Genotype::LMAN
N13::Genotype::Ear
N29::Genotype::SAM
N6::Genotype::Leaf
N9::Genotype::SAM
N28::Genotype::Root
N30::Genotype::Seed
N8::Genotype::Seedling
N3::Tissue::B73_ATLAS
N2::Tissue::B73_ATLAS
N45::RIL::W22xTeosinte
N5::Tissue::All_ATLAS
N4::Tissue::Seed_dev
N10::Genotype::Root
N14::Genotype::Root
N23::Genotype::L3Tip
N27::Genotype::Leaf
N12::Tissue*Genotype::5_tissues
N15::Genotype::Shoot
NES
2
1
0
−1

Figure S15.
